# Spatial Change of Dominant Baltic Sea Demersal Fish Across Two Decades

**DOI:** 10.1002/ece3.71309

**Published:** 2025-04-21

**Authors:** Liam MacNeil, Frane Madiraca, Saskia Otto, Marco Scotti

**Affiliations:** ^1^ Marine Ecology Research Division GEOMAR Helmholtz Centre for Ocean Research Kiel Kiel Germany; ^2^ Institute for Marine Ecosystem and Fisheries Science University of Hamburg Hamburg Germany; ^3^ Institute of Biosciences and Bioresources National Research Council of Italy Sesto Fiorentino Italy

**Keywords:** biogeography, biomass distribution models, hierarchical generalized additive models, large marine ecosystem

## Abstract

The range and biomass distribution of marine fish species offer insights into their underlying niches. Quantitative data are rare compared to occurrences and remain underused in species distribution models (SDMs) to explore realized niches—the actual space occupied by a species shaped by abiotic and biotic factors. Local densities drive differences in species contributions to ecological processes and ecosystem function rather than through presence alone. If a species growth rate is strongly controlled by macro‐environmental conditions, then predicting geographical abundance or densities should be possible. We collated 20 years (2001–2020) of standardized scientific bottom trawl data to fit several versions of hierarchical generalized additive models using biomass (kg km^−2^) of four dominant demersal species (Common dab, European flounder, European plaice, Atlantic cod) within yearly and seasonal (winter and autumn) time windows. Covariates were represented with trawl‐level geographic information (position, depth) and high‐resolution oceanographic features. This work illustrates species‐specific spatiotemporal biomass patterns across two decades and demonstrates superior predictive performance with seasonally variable smoothing terms, revealing seasonally different responses to oceanographic predictors. Firstly, we find relative stasis in Common dab biomass which is linked to the macro‐environmental salinity gradient in the western Baltic Sea but with different temperature responses across seasons. Secondly, we show both European flounder and plaice have increased in biomass in the western Baltic Sea with different seasonal relationships to bottom temperature, and that flounder switches between salinity conditions based on season during spawning/feeding periods. Lastly, both juvenile and adult Atlantic cod life stages are shown to have declined most significantly in the Bornholm Deeps and the Gdańsk Deeps. For cod, we conclude that biomass was less reliably predicted in comparison to the other major Baltic demersals studied here, warranting dynamic fishing covariates as a formerly major commercial fishing target. These models approach more dynamic species distribution models and are increasingly valuable to constrain uncertainties in biogeographic forecasting which often rely on annually‐averaged response curves, occurrence data, and suitability maps which rarely discriminate between areas of high and low biomass areas in space and time.

## Graphical abstract

This work maps demersal fish biomass in the Baltic Sea using hierarchical generalized additive models. It analyzes biogeographic patterns in this Large Marine Ecosystem, capturing broad spatiotemporal changes in biomass distributions, best explained by a combination of geographic and seasonal abiotic covariates.

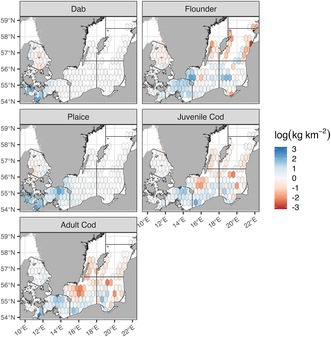

**Abbreviations**
BITS: Baltic international trawl survey
CMEMS: Copernicus marine environmental service
HGAM: hierarchical generalized additive models
MAE: mean absolute error
PSU: practical salinity units
SCOBI: Swedish coastal and ocean biogeochemical model
SDM: species distribution model

## 1 Introduction

The Baltic Sea is among the world's largest inland brackish seas (Leppäranta and Myrberg 2009). It exhibits relatively low genetic diversity of species (Johannesson and André 2006) and contains a species‐poor fish community that has been heavily relied upon and exploited by humans for centuries, analogous to other intensely exploited marginal seas (Jackson et al. 2001; Lotze et al. 2006). This shallow, human‐modified ecosystem underwent regime shifts driven by overfishing and eutrophication in the 1980–1990s (Österblom et al. 2007; Tomczak et al. 2013), and is pressingly vulnerable to anticipated climate and environmental changes (Reusch et al. 2018). The collapse of Eastern Baltic cod populations illustrates the ecological consequences of these complex dynamics, driven by a combination of low oxygen and salinity, high temperatures, nutrient runoff, and fishing pressure (Möllmann et al. 2009). The diminished role of cod as a Baltic Sea predator has been related to shifts in distribution and abundance in some flatfish considered as demersal (i.e., bottom‐associated) competitors (Orio et al. 2020; Haase et al. 2020). Flatfish represent a diverse assemblage within the Baltic fish community (Smoliński and Radtke 2017), are of high economic value (e.g., Hyytiäinen et al. 2021) and play a relevant ecological role linking upper to lower trophic levels (Scotti et al. 2022).

The contemporary geographic distributions of demersal fish species offer key insights into their environmental preferences, range limits, and determine the area within which biotic interactions occur (Guisan et al. 2013). As human‐driven environmental and climate changes are expected to alter these conditions further, understanding the drivers of species distributions and abundances or biomass is an urgent task (Urban et al. 2016). The controls over species distributions are related to a multi‐dimensional niche concept (Grinnell 1917; Hutchinson 1957). The fundamental niche is often conceived as the determining environmental factors in a species ability to persist under a range of environmental conditions; the realized niche combines abiotic and biotic factors, which influence a given species ability to establish and achieve positive growth rates (Godsoe et al. 2017). The relative importance of abiotic factors and biotic interactions in structuring species niches and resulting biogeography has been increasingly debated with larger empirical datasets and more powerful modeling tools (Wiens 2011; Pigot and Tobias 2013; Cadotte and Tucker 2017; Ovaskainen et al. 2017). Species distribution models (SDMs; also referred to as ecological niche models, see also Peterson and Soberón 2012) have been widely adopted in terrestrial and marine ecology to correlate occurrence data types (presence only, presence‐absence) to long‐term averaged climate conditions (Guisan and Thuiller 2005). As such, SDMs generally relate abiotic effects to species distributions (i.e., fundamental niche; Elith and Leathwick 2009), which have shown large effects beyond local scales (Soberón 2007; Thuiller et al. 2015; King et al. 2021) including across the west–east gradients of temperature and salinity in the Baltic Sea (Pecuchet et al. 2016). In the marine realm, SDMs have been used to assess species distributions across broad environmental gradients, invasion risk from alien species, and searching for effective ways to better inform conservation strategies (Robinson et al. 2017; Melo‐Merino et al. 2020). Accurately representing the niches and distributions of important fish species will help prioritize conservation efforts within the Baltic Sea, which displays unique eco‐evolutionary constraints between the North Sea–Baltic Sea transition zone (Johannesson et al. 2020) and is replete with complex international governance challenges (VanDeveer 2011).

Quantitative data based on species abundances or biomass are typically less widely available than occurrences (Hortal et al. 2015) and have remained underused to disentangle niche relationships (Howard et al. 2014; Rousseau and Betts 2022), especially in marine environments (Young and Carr 2015; Erauskin‐Extramiana et al. 2019; Waldock et al. 2022). Species make unequal contributions to ecological processes and ecosystem function through different local densities, rather than through presence alone (Ehrlén and Morris 2015), and quantitative‐based measures can represent an important signal of population growth or collapse (Ceballos et al. 2020). However, abundance patterns do not always appear strongly correlated to species‐environment suitability estimates from occurrence‐based SDMs (VanDerWal et al. 2009; Weber et al. 2017; Dallas and Hastings 2018). If a species growth rate is strongly controlled by macro‐environmental conditions and affected by steep environmental gradients, for which there is strong evidence to be acting on the western and central Baltic Sea demersal fish community composition (Pecuchet et al. 2016; Frelat et al. 2018), then predicting geographical abundance or biomass density patterns should be possible (Waldock et al. 2022).

In this work, we applied three versions of hierarchical generalized additive models (HGAMs) to predict biomass densities (kg km^−2^) of four demersal fish species (Common dab, European flounder, European plaice, and Atlantic cod) in the Baltic Sea. Fish biomass densities were derived from internationally coordinated, biannual (first and fourth quarter) fisheries‐independent trawl data covering 2001–2020 and differentiated by ontogenetic size classes for Atlantic cod. The HGAMs were constructed to fit and predict biomass densities at yearly and seasonal (biannual) time windows, with each successive model version incorporating greater complexity in predictor‐response relationships, building from solely geographic predictors to include seasonal abiotic (sea bottom temperature, salinity, and oxygen) predictors. The HGAM versions which combined geographic and seasonal abiotic predictors offered the highest performing models, clarifying seasonal differences in species‐specific relationships to environment and predicting spatiotemporal changes in species biomass densities. Accurately summarizing the changing geographic distribution of species densities across highly mobile fish groups could help refine predictions of future shifts under environmental and climate changes.

## 2 Methods

### 2.1 Study Region

**FIGURE 1:**
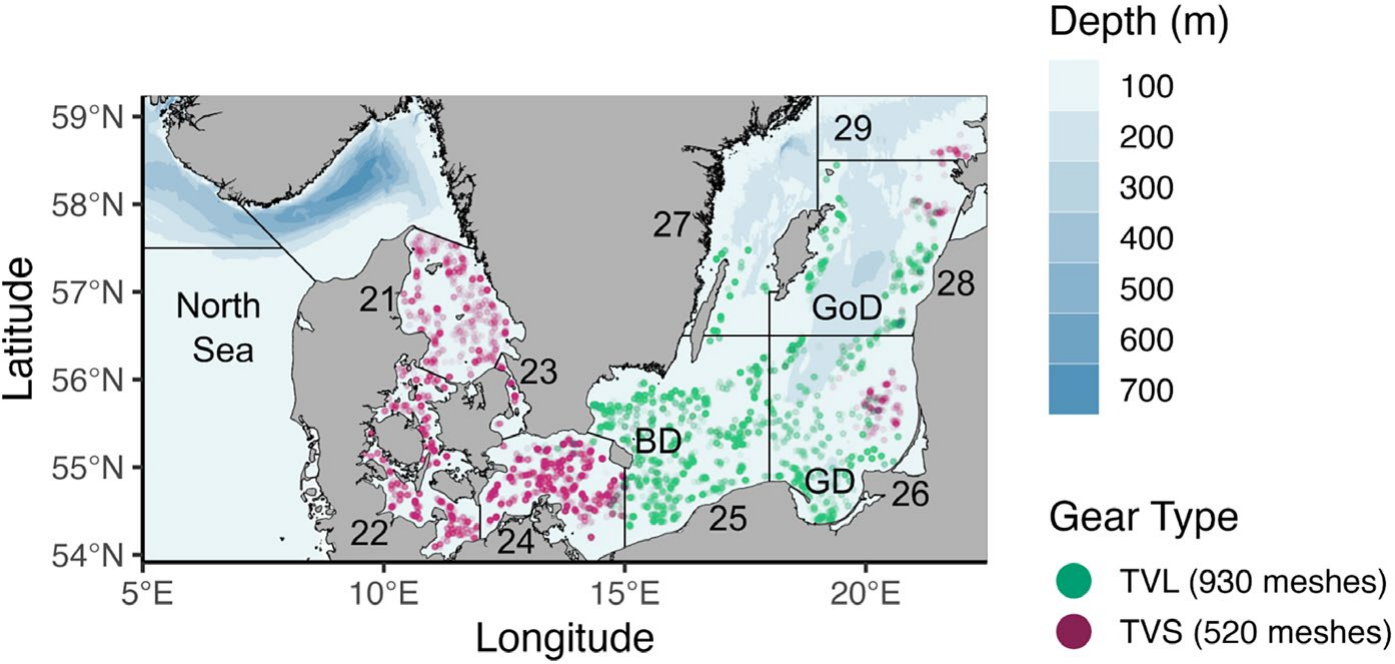
The Baltic Sea study region on the European shelf and linked to the North Sea; sampling points are colored by gear size/type overtop bathymetry (Weatherall et al. 2015). The labeled ICES subdivisions (21–29) delimit the boundary area, and deep basins are annotated, including Bornholm Deeps (BD), Gdańsk deeps (GD) and Gotland deeps (GoD).

The Baltic Sea extends from the northeast Atlantic Ocean shelf into northern Europe and is characterized by strong gradients of salinity and species diversity (Leppäranta and Myrberg 2009). The study region includes ICES subdivisions 21–29 covering the majority of the Baltic Sea, ranging from the marine‐brackish transitions zones (Kattegat, Øresund, and western Baltic Sea) to the Baltic Proper (Arkona Basin, eastern Bornholm Deeps, and eastern Gotland Basin). Seafloor depth is generally shallow (< 50 m); three prominently deep areas include Bornholm Deeps (> 70 m), Gdańsk Basin (> 100 m), and eastern Gotland Basin (> 175 m; Figure 1). Each of these regions represents important spawning and nursery grounds for many demersal fishes (HELCOM 2021).

### 2.2 Observational Data

The Baltic International Trawl Survey (BITS) is a multi‐decadal program collecting standardized, fisheries‐independent demersal trawls (ICES 2017). Surveys are regularly biannual, occurring in Q1 (Feb–Mar; winter) and Q4 (Oct–Nov; autumn) using standardized gears (TVL 930 meshes, TVS 520 meshes) at randomly stratified stations based on ICES subdivisions (Figure 1). Sampling design is described in more detail within ICES protocols (ICES 2017). We extracted BITS exchange data (haul and catch information) covering biannual trawls between 2001 and 2020, chosen as the beginning of international gear standardization (ICES 2017) and to match the time window available for our environmental covariates. Observations corresponding to negative bottom oxygen values—produced to represent hydrogen sulfide concentrations in the SCOBI model (Eilola et al. 2009)—were removed as they represented rare (< 1.3%) extremes in the oxygen data. All subsequent analyses were conducted in the R programming language (R Core Team 2022). From the full exchange data, hauls were removed which: (1) Did not record haul duration, longitude, or latitude, (2) were not designated as “valid” (< 15 or > 90 min) or “additional” (3) did not record species length class, or (4) did not record catch weight (kg). The four dominant demersal fish species in the Baltic Sea focused on here include Atlantic cod ( *Gadus morhua* ), European plaice ( *Pleuronectes platessa* ), European flounder ( *Platichthys flesus* ), and Common dab ( *Limanda limanda* ). We note, however, that a distinct flounder species (*Platichthys solemdali*) predominates towards the northern Baltic proper (Momigliano et al. 2018), but our limited trawl coverage in these areas suggests European flounder constitute the largest fraction of our observations. For all hauls, zeros were imputed where these species were not recorded, creating a zero‐inflated dataset (22.1%) familiar to many ecological studies (Martin et al. 2005). The final dataset contains 8991 individual trawls, averaging 450 trawls per year (Figures S1–S5). We standardized catch weight by total swept area (km^2^) as a measure of effort (ICES 2019) and the unit for modeling represents a measure of species‐specific biomass density (kg km^−2^; hereafter “biomass”).

### 2.3 Covariates

Environmental layers were collected to represent oceanographic conditions in the Baltic Sea at bottom depths (Table 1). Oceanographic variables derived from high‐resolution (~2 nautical miles) physics and biogeochemistry reanalysis products of the NEMO‐Nordic ice‐ocean model coupled to the Swedish Coastal and Ocean Biogeochemical (SCOBI) model (Eilola et al. 2009; Liu et al. 2017) available in Copernicus Marine Environmental Service (CMEMS).^1^ All oceanographic variables were downloaded as monthly means. To capture seasonal patterns and better match environmental conditions during trawls, each variable was averaged grid‐cellwise by values within the first (Q1; winter) and fourth (Q4; autumn) quarters of each year, weighted by the proportion of trawl observations of each month. We selected abiotic variables at the sea bottom (temperature, oxygen, salinity) from each model as they shape the ecology and distribution of demersal fishes in the Baltic Sea (Smoliński and Radtke 2017; Rau et al. 2019; Orio et al. 2019; Brander 2022; see also Table 1). In preparation for modeling, trawl data were spatially thinned to covariate resolution (~2 nautical miles) to reduce overfitting due to sampling bias of repeated observations (Boria et al. 2014). Trawl data were then geographically matched to covariates.

**TABLE 1:** Environmental covariates (predictors) overview for HGAMs, including their expected impacts on demersal fish biomass supported with example references.

Variable	Unit	Source	Data product	Expected effect
Sea bottom temperature	Potential temperature (°C)	CMEMS	Physics reanalysis	Somatic growth and recruitment rates (Olsson et al. 2012)
Sea bottom salinity	PSU (‰)	CMEMS	Physics reanalysis	Osmoregulation stress (Kültz 2015); egg buoyancy and larval mortality (Nissling and Westin 1997)
Sea bottom oxygen	Dissolved O_2_ concentration (mg L^−1^)	CMEMS	Biogeochemistry reanalysis	Feeding rates and somatic growth (Brander 2022)

### 2.4 Hierarchical Generalized Additive Models (HGAMs)

We fit hierarchical generalized additive models (HGAMs; Pedersen et al. 2019) onto the spatiotemporal dataset of demersal fish biomass (kg km^−2^) to test the importance of geography and dynamic, bottom‐associated abiotic variables (temperature, oxygen, and salinity) in predicting different species biomass distributions. We split predictions in hierarchical species groups, with an ontogenetic divide between adult (≥ 35 cm) and juvenile (< 35 cm) cod (Valentinsson et al. 2019). HGAMs were fit in *mgcv* (Wood 2017) with the *bam* function using fast restricted maximum likelihood (fREML) with discretized covariates to speed model fitting. To produce parsimonious model versions, variable selection included null space penalization for each smoothing term, penalizing non‐informative variables towards zero. Model diagnostics were inspected with mgcv functions *gam.check* for model residuals (Figure S6) and *concurvity* as a generalized indication of collinearity in model terms (Dormann et al. 2013), which remained below 0.8 (Leonardi et al. 2022; Figure S7) and GAMs show robust performance despite concurvity (Wood 2008). Due to the zero‐inflated nature of the dataset, biomass values for the *i*th observation for species group *j* $(B_{\mathrm{ij}}$) were modeled using a Tweedie distribution with a log‐link function. The $\mu_{\mathrm{ij}}$ represents the mean biomass value for species *j* at a particular time (year and season) and location for the *i*th observation. The $\phi_{j}$ and $\rho_{j}$ represent a dispersion and power parameter, respectively, that modify the mean–variance relationship for each species *j* in the Tweedie distribution. An error term was assumed for each model version ($\epsilon_{i}$).

(1) $$B_{\mathrm{ij}}\sim \text{Tweedie}\left(\mu_{\mathrm{ij}},\phi_{j},\rho_{j}\right)$$

HGAM version‐I (Equation 2) includes a random intercept ($\beta_{\text{season}}$) by season with geographic predictors for expected mean species biomass, log transformed, combining into a spatiotemporal term a tensor product factor smooth (bs = “fs”) between spatial position (longitude, latitude) and year encoded as a factor (e.g., Zabihi‐Seissan et al. 2024), with unique smoothers per species (by = *species*_*j*_). To incorporate variations by depth, we add a tensor product smooth for the interaction between depth and year (continuous) with species‐specific smoothers.

(2) $$\begin{array}{c} \log\left(\mu_{\mathrm{ij}}\right)=\beta_{\text{season}}+\mathrm{ti}\left({\text{latitude}}_{i},{\text{longitude}}_{i},{\text{year}}_{i},\mathrm{by}={\text{species}}_{j}\right)\\ +\;\mathrm{te}\left({\text{depth}}_{i},{\text{year}}_{i},\mathrm{by}={\text{species}}_{j}\right)+\epsilon_{i} \end{array}$$

HGAM version‐II (Equation 3) builds on version‐I, including the primary spatiotemporal term of position and year varying by species, adding species‐specific smoothers between depth and each abiotic covariate (temperature, oxygen, salinity)—we found repeating the $\mathrm{te}\left({\text{depth}}_{i},{\text{year}}_{i},\mathrm{by}={\text{species}}_{j}\right)$ term with abiotic covariates produced almost complete concurvity (> 0.96). The separate smoothers allowed group‐level variations to each covariate using thin plate regression splines (bs = “tp”).

(3) $$\begin{array}{c} \log\left(\mu_{\mathrm{ij}}\right)=\beta_{\text{season}}+\mathrm{ti}\left({\text{latitude}}_{i},{\text{longitude}}_{i},{\text{year}}_{i},\mathrm{by}={\text{species}}_{j}\right)\\ +\;s\left({\text{depth}}_{i},\mathrm{by}={\text{species}}_{j}\right)+s\left({\text{temperature}}_{i},\mathrm{by}={\text{species}}_{j}\right)\\ +\;s\left({\text{oxygen}}_{i},\mathrm{by}={\text{species}}_{j}\right)+s\left({\text{salinity}}_{i},\mathrm{by}={\text{species}}_{j}\right)+\epsilon_{i} \end{array}$$

HGAM version‐III (Equation 4) extends the relationship to the environment further to permit seasonally independent responses to covariates and different smoothing penalties by species. For model versions‐II and ‐III, we penalize the square second derivative (*m* = 2) of group‐level smoothers to limit excessive curvature (Pedersen et al. 2019). Note that we exclude seasonal random smoothers in version‐III for the intercept, as these are included in the factor smooths for depth, temperature, oxygen, and salinity.

(4) $$\begin{array}{c} \log\left(\mu_{\mathrm{ij}}\right)=\mathrm{ti}\left({\text{latitude}}_{i},{\text{longitude}}_{i},{\text{year}}_{i},\mathrm{by}={\text{species}}_{j}\right)\\ +\;s\left({\text{depth}}_{i},\text{season},\mathrm{by}={\text{species}}_{j}\right)\\ +\;s\left({\text{temperature}}_{i},\text{season},\mathrm{by}={\text{species}}_{j}\right)\\ +\;s\left({\text{oxygen}}_{i},\text{season},\mathrm{by}={\text{species}}_{j}\right)\\ +\;s\left({\text{salinity}}_{i},\text{season},\mathrm{by}={\text{species}}_{j}\right) \end{array}$$

The default values for the number of basis functions in smoothers (*k*) was chosen, maximizing the allowed degrees of freedom (*k*−1) and for greater variation in individual smoothers. This was decided as even substantially larger effective degrees of freedom (EDF) compared to *k* (e.g., EDF_ti(lon,lat,year)_ = 124 vs. *k*′ = 50) remained indicated as problematically significant, implying an inadequate number of basis functions in *gam.check*. Despite this, changing *k* values for individual variables left the diagnostic plots and partial dependence curves unchanged, thus we maintained *k* values to a default (Table S1). To inspect the apparent issue of basis dimension, we plotted model residuals against each covariate for each model version (Figures S8 and S9) and did not find any clear residual dependence to geographic, temporal, or environmental structure. We note here that the *gam.check* output is a heuristic, and this apparent significance could be due to missing variables which species biomass might exhibit a dependence (e.g., spatially‐resolved biotic interactions). Trawl observations are clustered in time (year and season), leading us to incorporate an AR1 autocorrelation matrix through the rho parameter in the bam function. The value of rho was determined for each model form (version I = 0.17, II = 0.21, III = 0.15) using the R package *itsadug* (Van Rij et al. 2015).

### 2.5 Model Evaluation and Prediction

Models were assessed through k‐fold cross validation, with data split into train‐test k‐folds (*k* = 10) for fitting and evaluation, respectively (Figure S10). Model performance metrics compared observed versus predicted biomass using three regression metrics presented in Waldock et al. (2022): Discrimination (Pearson's correlation coefficient), accuracy (Mean Absolute Error; MAE), and dispersion (*σ*_est_/*σ*_obs_)^2^. After model evaluation under cross validation, final predictions for mapping incorporated all observations (Step 5, option 1 in Roberts et al. 2017), favoring prediction quality over error estimation although we expect reasonable error estimates from cross validation. We visualize predicted species biomass patterns as the difference in average biomass within 0.5° grid cells between the last 5 years (2016–2020) and the first 5 years (2001–2005) of the trawl survey. Together with the regression metrics, which illustrate an individual model's predictive ability, we also compared model parsimony using AIC (Akaike's information criterion; Akaike 1974) of final models used for prediction mapping and generated partial dependence curves to assess the relationship of each species to geographic and abiotic covariates using the R package *gratia* (Simpson 2018). All modeling specifications are detailed in Supporting Information including R script for replication with an ODMAP protocol (Appendices S1 and S2; Zurell et al. 2020).

## 3 Results

### 3.1 Models Performance

**FIGURE 2:**
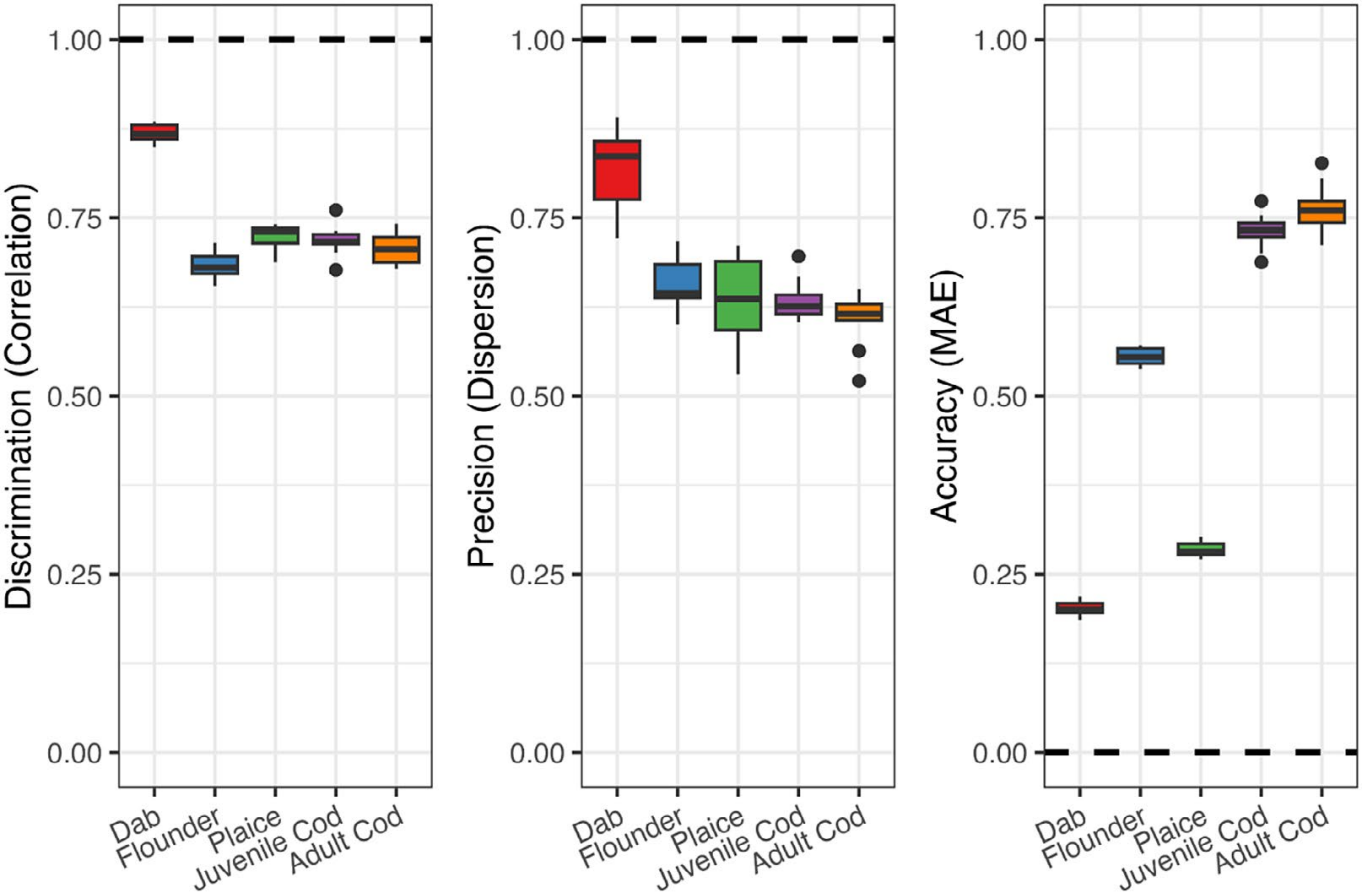
Regression metrics of HGAM version‐III performance, including geographic and seasonal abiotic predictors, based on discrimination (Pearson's correlation coefficient), accuracy (mean absolute error; MAE), and precision (ratio between variance of estimated and observed biomass density). Boxplot central lines indicate median values and boxes' interquartile range symbolizes 25th and 75th percentiles. Dotted lines for each panel mark the respective optimal metric values.

Model predictions measured on k‐fold cross validation indicated relatively similar discrimination ($\bar{x}$_*r*_ = 0.71 ± 0.14), precision ($\bar{x}$_*σ*_ = 0.59 ± 0.24), and accuracy ($\bar{x}$_MAE_ = 0.51 ± 0.14) between model versions, but we did observe stepwise improvement by including abiotic covariates and at seasonal resolution. The highest predictive performance for every metric was achieved by HGAM version‐III, including seasonal abiotic covariates. Individual performance metrics for each model and time period are shown in Figure 2 and Table S2. Across species, Common dab biomass was uniformly predicted with the greatest discrimination, accuracy, and precision. Secondarily, European plaice and flounder were predicted with similar discrimination and precision, but plaice displayed greater MAE in biomass predictions. Both juvenile and adult cod were similar to flounder and plaice in terms of biomass discrimination and precision, but rarely predicted at observed values (high MAE). A similar stepwise gain in model parsimony was found by comparing AIC, with the lowest values estimated for model version‐III, including seasonal abiotic covariates (Table S2).

### 3.2 Seasonal Partial Effects

**FIGURE 3:**
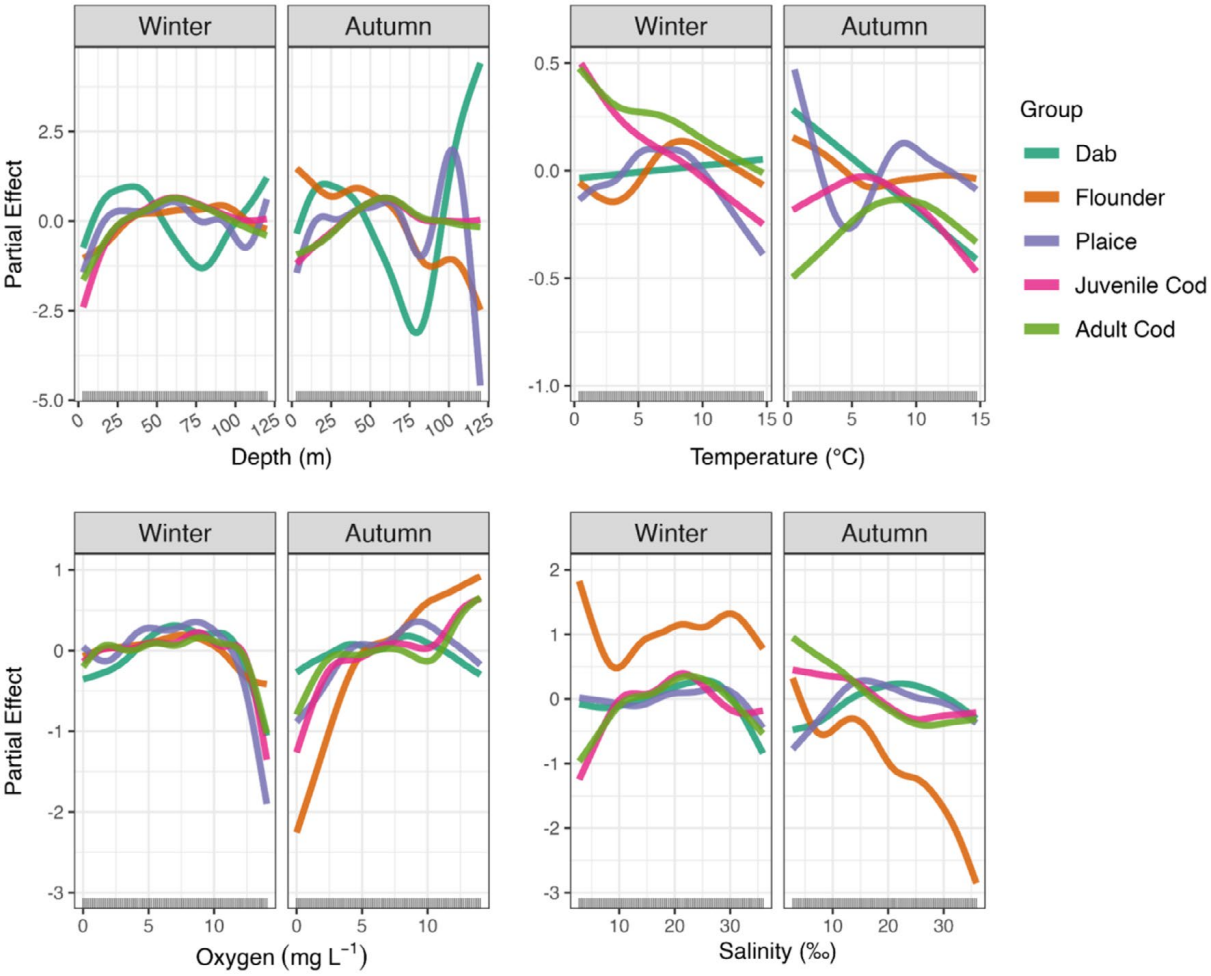
Partial dependence curves generated from HGAM version‐III, including geographic and seasonal abiotic (sea bottom) predictors. For each variable, species‐level random smoothers allow individually unique penalties (wiggliness) and independent shapes by season. Rugs mark the observed data coverage on the *x*‐axis for each covariate.

Assessing the partial effects on the mean response of demersal fish biomass in the Baltic Sea indicates values can differ considerably across depths and oceanographic conditions, and by season (Figure 3). The spatiotemporal partial effects are shown by species in Appendix S1 (Figures S11–S16). By depth, mean demersal biomass generally responded positively to deeper waters up to 50–75 m, including in HGAM version‐II, capturing strong effects of the shallower western Baltic Sea region (ICES subdivisions 21–24; Figure S17). Dab and flounder were the notable exceptions, declining in biomass towards 75 m most strongly in autumn. The abiotic response curves generally differed by season and species, with bottom temperatures producing the greatest differences. Dab displays a positive relationship to temperature in winter and a negative trend in autumn. Flounder showed higher partial effects at bottom temperatures ~8°C–11°C in winter and only positive partial effects < 5°C in autumn, whereas plaice shows relatively higher average biomass at temperatures between 5°C and 10°C in winter and responds variably across a wide temperature range in autumn. Both juvenile and adult cod vary similarly to bottom temperatures, with declining average biomass at higher temperatures in winter and with generally higher biomass at temperatures up to ~9°C in autumn. Species tend to respond positively to increasing bottom oxygen concentrations in both seasons; however, each declines in biomass at the highest concentrations > 10 mg L^−1^. The partial effects for bottom salinity are generally positive in winter for all species between 10 and 30 PSU, but this relationship flips in autumn with higher partial effects for flounder and cod in fresher conditions (< 10 PSU). In autumn, these relationships change most strongly in flounder, which decline monotonically in average biomass at higher salinities.

### 3.3 Spatiotemporal Patterns of Species Biomass

**FIGURE 4:**
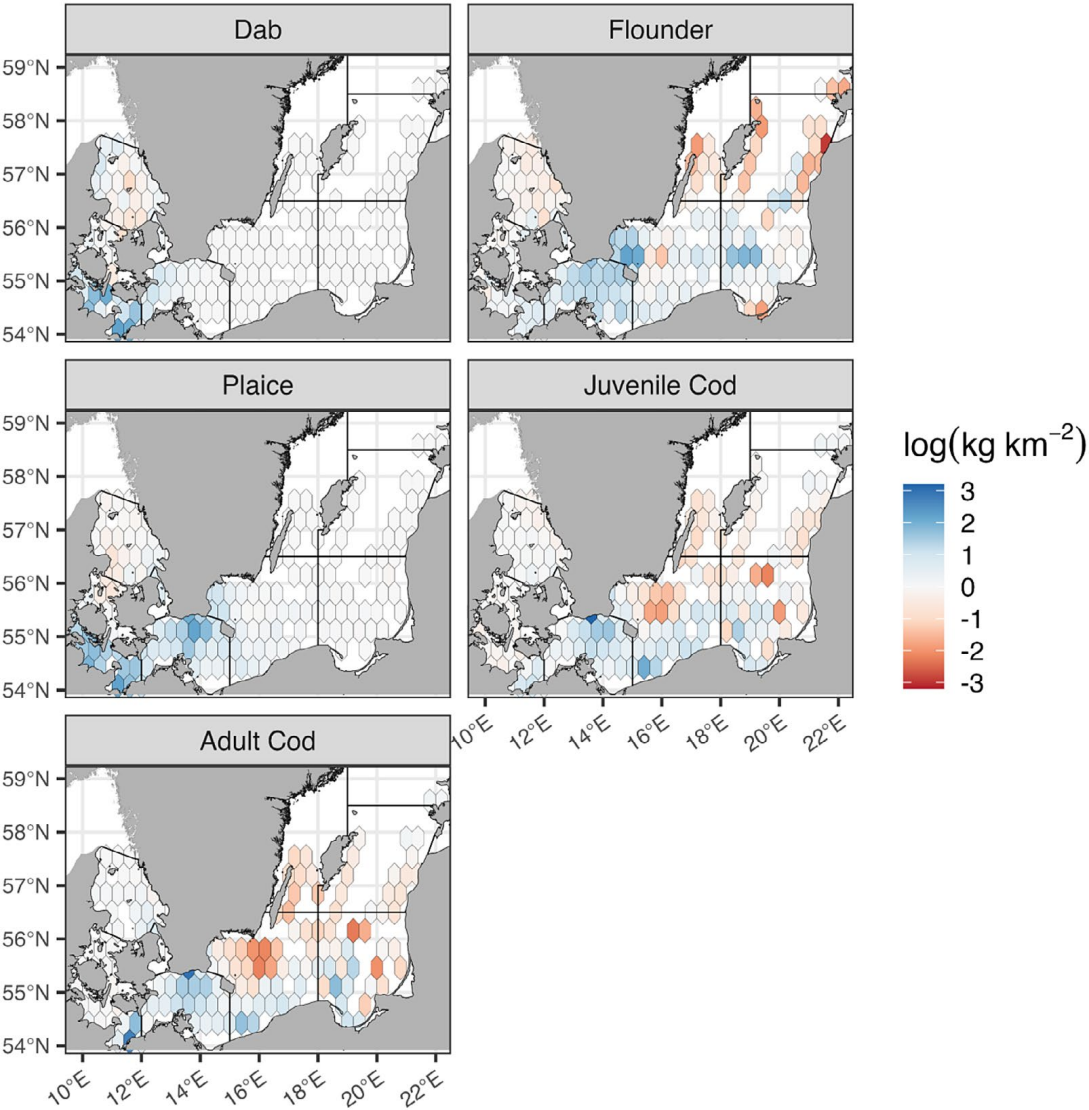
Species biomass predictions, expressed as log(kg km^−2^), calculated as the difference in mean grid cell biomass between the last 5 years (2016–2020) and the first 5 years (2001–2005) of the trawl survey.

Spatiotemporal patterns in species biomass predictions were broadly species specific in our HGAMs and are shown as the total change in biomass per grid cell in Figure 4. Yearly species biomass predictions are shown in Figures S18–S22. First, Common dab shows little discernible spatial changes in biomass, instead characterized by relative stasis and with minor biomass fluctuations confined to the western Baltic Sea. Second, European flounder increased widely across the western Baltic Sea and relative declines appear in the northern Baltic proper—however, we expect these apparent declines to have been influenced by unequal sampling coverage in the earliest years of the trawl survey (Figure S19). Third, European plaice have consistantly increased in biomass throughout the western Baltic Sea towards present. Fourth, both juvenile and adult cod declined in biomass in several deeper regions in the Baltic Sea: Both cod life stages declined in the Bornholm Deeps, most substantial among juveniles, and near the Gdańsk Deeps where adults showed the greatest declines in biomass. Outside these deeper areas and the northern Baltic proper, both cod life stages show some relative increases in biomass.

## 4 Discussion

This work depicts changing spatial patterns of demersal fish biomass and offers descriptions of their seasonal relationship to abiotic conditions throughout the past two decades in the Baltic Sea. Atlantic cod is arguably the most widely studied species in the Baltic Sea (Hüssy 2011; Neuenfeldt et al. 2020; Svedäng et al. 2022). Our model results confirm and expand previously described spatiotemporal patterns, based on similar trawl data, of cod in the eastern Baltic Sea: A theme of spatial contraction and quantitative decline in juveniles and adults towards present (Casini et al. 2019; Orio et al. 2019). This work clearly maps a striking decline of juvenile and adult cod life stages from nursery areas near the Gdańsk deeps and the erosion of biomass in spawning areas of the Bornholm Deeps compared to the early 2000s—a period already following precipitous declines in cod populations in the Baltic during the 1980s–1990s (Möllmann et al. 2021; Eero et al. 2023). These spatiotemporal predictions align with related models of Eastern Baltic cod biomass declines in the 1990s, which were accompanied by widespread depreciations in cod body index and highly concentrated near the Bornholm Deeps (Lindmark et al. 2023).

Across flatfishes, the relative stasis of dab in space reflects average patterns where biomass is strongly filtered by the salinity gradient and restricted to Kattegat, Øresund, and parts of the Danish Straits in the western Baltic Sea (Heessen et al. 2015). Declines of flounder biomass in the northern Baltic proper are a more recent trend of the 2010s but are exaggerated by limited coverage by BITS in the early 2000s (Figure 4; Figure S19). However, compared to 1980–1990s, biomass appears to have increased substantially (Orio et al. 2019)—caveated by potential catchability biases in different gear standardizations prior to 2001 (Orio et al. 2017).^3^ The similar shifts of plaice and flounder, increasing in the western Baltic Sea towards present, were clearly not limited by a similar environmental filter as dab, reinforcing important questions about the role of increased competition by demersal fishes contributing to the continued decline of cod populations (Orio et al. 2020). By comparison, springtime trawl data from 2004 to 2016 in the western Baltic Sea indicate that missing biotic variables relevant to flatfish biomass include quantitative distributions of food sources such as arthropods, annelids, and mollusks (Rau et al. 2019). This limit to our study is in part compensated through bottom oxygen concentrations, the availability of which is a prerequisite for the growth of demersal food sources (Villnäs et al. 2012). Based on the performance of our best model version incorporating seasonal abiotic covariates (HGAM version‐III), cod is less reliably predicted than the other major demersals considered here; we expect that, as a formerly major commercial target in the Baltic, dynamic fishing effort or nominal catch data are needed at relatively fine resolution (e.g., downscaled from global estimates Rousseau et al. 2024) to incorporate the negative impacts particularly on cod biomass.

Driven by the strong environmental gradients in the Baltic Sea, particularly salinity, population differentiation in multiple fish species has emerged with strong geographic clustering (Johannesson et al. 2020). This creates relevant physiological and thus niche differences that generate intraspecific variation (Smith et al. 2019) and yet clear geographic divides rarely exist to split data by subpopulations (Zhang et al. 2021). So, despite the richness of the multi‐decadal trawl survey presented here, important subpopulation factors are missing in our fish groups: At least two cod stocks (East and West) in the Baltic Sea bear different niche breadth in their tolerances to salinity (e.g., 14–36 PSU) and temperature (Righton et al. 2010), and at least one example of mutation in hemoglobin proteins appears adaptive to different temperature and oxygen conditions (Andersen et al. 2009). In space, these niche differences can filter larvae through differences in neutral buoyancy as western Baltic cod eggs sink at lower salinities (< 20 PSU, Nissling and Westin 1997), becoming exposed to hypoxic waters < 2 mL L^−1^ O_2_. But a definitive geographic boundary between subpopulations is complicated by population mixing in the Arkona Basin (ICES subdivision 24) recorded independently from otoliths (Hüssy et al. 2016) and single nucleotide polymorphisms (SNPs; Weist et al. 2019). A combination of trawl data, otoliths, and genomic microsatellites deepens the degree of coexistence in the Arkona Basin, confirming population overlap in space and partitioned by depth, with western stocks occupying shallower zones and the eastern stock predominating in deeper waters (Schade et al. 2022). Similarly, European ( *P. flesus* ) and Baltic flounder (*P. solemdali*) display different adaptations to salinity, oxygen, and temperature—although our trawls rarely cover areas dominated by Baltic flounder, with relatively few observations surrounding Öland and Gotland in the Baltic proper (Momigliano et al. 2018). We expect the plaice distributions described here to include two major stocks: roughly, with a western stock predominating throughout Kattegat, Øresund, and the western Baltic Sea, while the second stock prevails in the Baltic proper (Ulrich et al. 2013). More broadly, limitations in observation data to resolve subpopulations with relevant niche differences persist across the SDM literature (Meynard et al. 2019). These aspects limit the generality of our HGAM descriptions and suggest improvement of species range forecasting under climate change with evolutionary markers (Aguirre‐Liguori et al. 2021); yet these data remain sparse in comparison to records of species occurrence or abundances.

Despite these limits, we resolve seasonal differences that arguably capture important life histories. Flounder biomass responded positively at higher salinities (> 10 PSU; Feb–March), just prior to or during spawning (Orio et al. 2017), but this relationship flips during autumn (October–November), likely reflecting seasonal feeding periods when both European and Baltic flounder appear to use shallow, fresher coastal waters prior to late autumn/early winter spawning migrations (Nissling and Dahlman 2010; Heessen et al. 2015). For dab and plaice, we build on work previously focused on the German sections of the BITS survey (Rau et al. 2019), generally showing that the estimated partial effects, unsurprisingly, do not hold across seasons and that species occupy different ranges of their realized niches. This is shown also in the apparent flip of the relationship between dab biomass and bottom temperatures between winter and autumn. For cod, it appears that the biomass of juveniles and adults responds similarly against temperature, oxygen, and salinity conditions but at different magnitudes. But these apparent differences can result in substantial spatiotemporal variation in larvae survival (Huwer et al. 2014), and local populations could exhibit distinct environmental preferences within the general patterns shown here. A key takeaway from this work is the species‐specific, seasonally variable response curves. These data can be repurposed as inputs to help parametrize and constrain uncertainties in distribution forecasting under scenarios of climate change or fishing pressures (Nascimento et al. 2023), instead of fixed annual or long‐term average response curves derived from occurrence data and suitability maps.

This work's mapping of species biomass consists of an interpolation within a well sampled range. However, model transferability (i.e., extrapolation beyond training region or timeframe), which indicates the generality of any model's representation of ecological mechanisms, remains a challenge to demonstrate for large‐scale models (Heikkinen et al. 2012; Yates et al. 2018). Limited transferability in SDMs is associated with poor representation of species underlying biology and ecology (Lee‐Yaw et al. 2022). The characteristics of transferable models or species traits that affect transferability in 129 North American bird species were explored by Rousseau and Betts (2022), revealing widespread failures in spatial transferability to predict species abundances and as a result, cautioning extrapolation beyond trained areas. We attempted to constrain model overfitting through spatial thinning, k‐fold cross validation, and by focusing on core abiotic variables (e.g., temperature, salinity, oxygen) which have causal links to species distributions and achieved biomass (Wenger and Olden 2012). Future work on transferability in SDMs will also benefit from testing scale (i.e., grid size) sensitivity of predictors and response variables (Lu and Jetz 2023). Altogether, applying quantitative data as a response variable such as our trawl biomass data, where sufficiently available, could provide greater model discrimination ability than typical occurrence data through a closer link to underlying processes (e.g., growth, mortality, competition) and thus increase transferability and biological realism (Waldock et al. 2022; Rousseau and Betts 2022). Towards that aim, we observe that our HGAMs can reliably predict biomass patterns which have been individually documented in past studies, but generally underpredict biomass, a common feature to many ecological models applied across large spatial or temporal extents (Waldock et al. 2022; Palacio and Clark 2023).

As a Large Marine Ecosystem (Sherman 1991), the Baltic Sea is among the best studied marine regions, which afforded our work unique access to fisheries‐independent trawling data that are consistent, standardized, and comparable across sites and years. However, better coverage of shallow (< 20 m) coastal regions could improve detection and understanding of population dynamics in critical spawning and nursery areas, especially in the western Baltic Sea (Kraufvelin et al. 2018). Sample coverage can be expanded to new sites or understudied depths by combining scientific surveys with commercial trawl data, but this presents further challenges of data integration: In the western Baltic Sea, scientific trawls are comparatively brief (less than 1 h) whereas commercial trawls can run up to 9 h (Rufener et al. 2021). Expanding and improving trawl data collection in the Baltic Sea can support more finer scale, temporally dense observations to underpin seasonal or monthly response curves for a wider variety of fish species, aiming to disentangle the local and regional outcomes which might be likely under the pressing environmental changes bearing on the Baltic Sea.

## Author Contributions

**Liam MacNeil:** conceptualization (lead), data curation (lead), formal analysis (lead), writing – original draft (lead), writing – review and editing (lead). **Frane Madiraca:** conceptualization (supporting), formal analysis (supporting), writing – review and editing (supporting). **Saskia Otto:** conceptualization (supporting), writing – review and editing (supporting). **Marco Scotti:** conceptualization (supporting), data curation (supporting), funding acquisition (lead), methodology (supporting), project administration (lead), writing – review and editing (supporting).

## Disclosure

The authors have nothing to report.

## Conflicts of Interest

The authors declare no conflicts of interest.

## Supporting information


Appendix S1.

Appendix S2.

## Data Availability

All data relevant to this analysis are freely available: DATRAS‐BITS (Baltic International Trawl Survey) exchange data and swept‐area assessment (https://datras.ices.dk/data_products/download/download_data_public.aspx). Copernicus marine service environmental layers. Baltic Sea Physics Reanalysis: https://doi.org/10.48670/moi‐00013. Baltic Sea Biogeochemistry Reanalysis: https://doi.org/10.48670/moi‐00012.

## Endnotes

^1^

https://marine.copernicus.eu/. Accessed May, 2022.

^2^
Variance of estimated (*σ*_est_) and observed (*σ*_obs_) biomass density.

^3^
In this study, the standardized gears help limit the effects of biased detection across years/seasons. The catchability bias is assumed to be constant (and we assume is reduced significantly by using constant gears), which is an oversimplification, albeit a widespread and useful one (Dean et al. 2021). We have emphasized throughout the value of gear standardization in our analysis and how it guided our choice of time period for modelling. Catchability estimates are used in estimating swept area (effort) by ICES. What the reviewer appears to be recommending, however, are additional estimates of catchability or multiple methods of pseudo‐absence generation to simulate the effects of absences in driving biomass patterns. Regionally‐specific catchabilities are rare (e.g., for 75 species in North Sea Walker et al. 2017) and in addition, we tested numerous pseudo‐absence generation steps during revisions of this journal‐ which were ultimately removed at the suggestion of the reviewers.
