# Supplementary material for: Spatial Change of Dominant Baltic Sea Demersal Fish Across Two Decades
Source: Ecol Evol. 2025 Apr 21;15(4):e71309. doi: 10.1002/ece3.71309 (PMC12011422; doi:10.1002/ece3.71309)
Supplement: Supplementary file 1 — Appendix S1. [file ECE3-15-e71309-s002.docx]

**Appendix S1:**

**Spatial change of dominant Baltic Sea demersal fish across two decades**


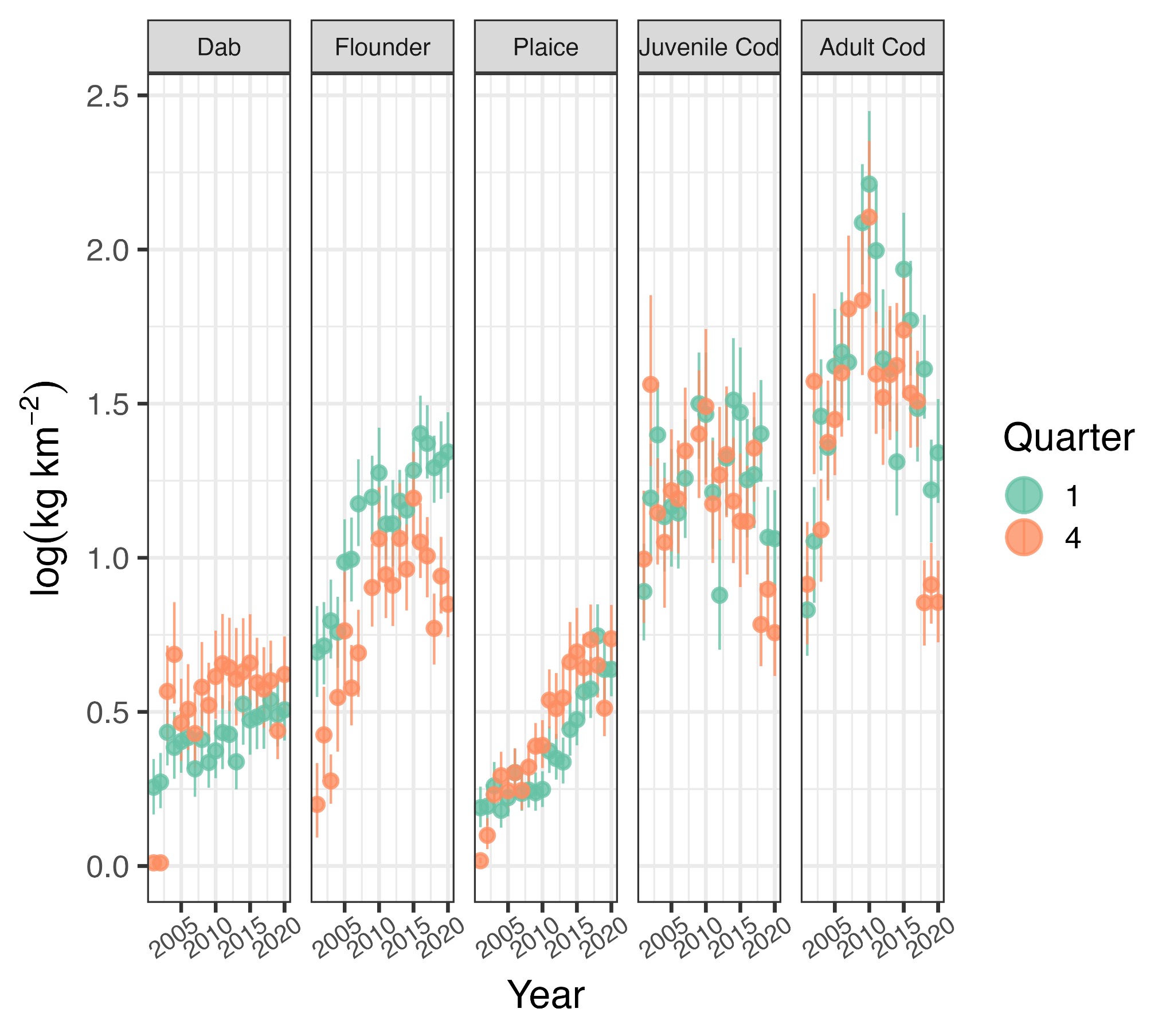


Figure S1. Trawl biomass densities throughout the 20-year time series as bootstrap means with 95% confidence intervals, colored by yearly quarter and faceted by species.


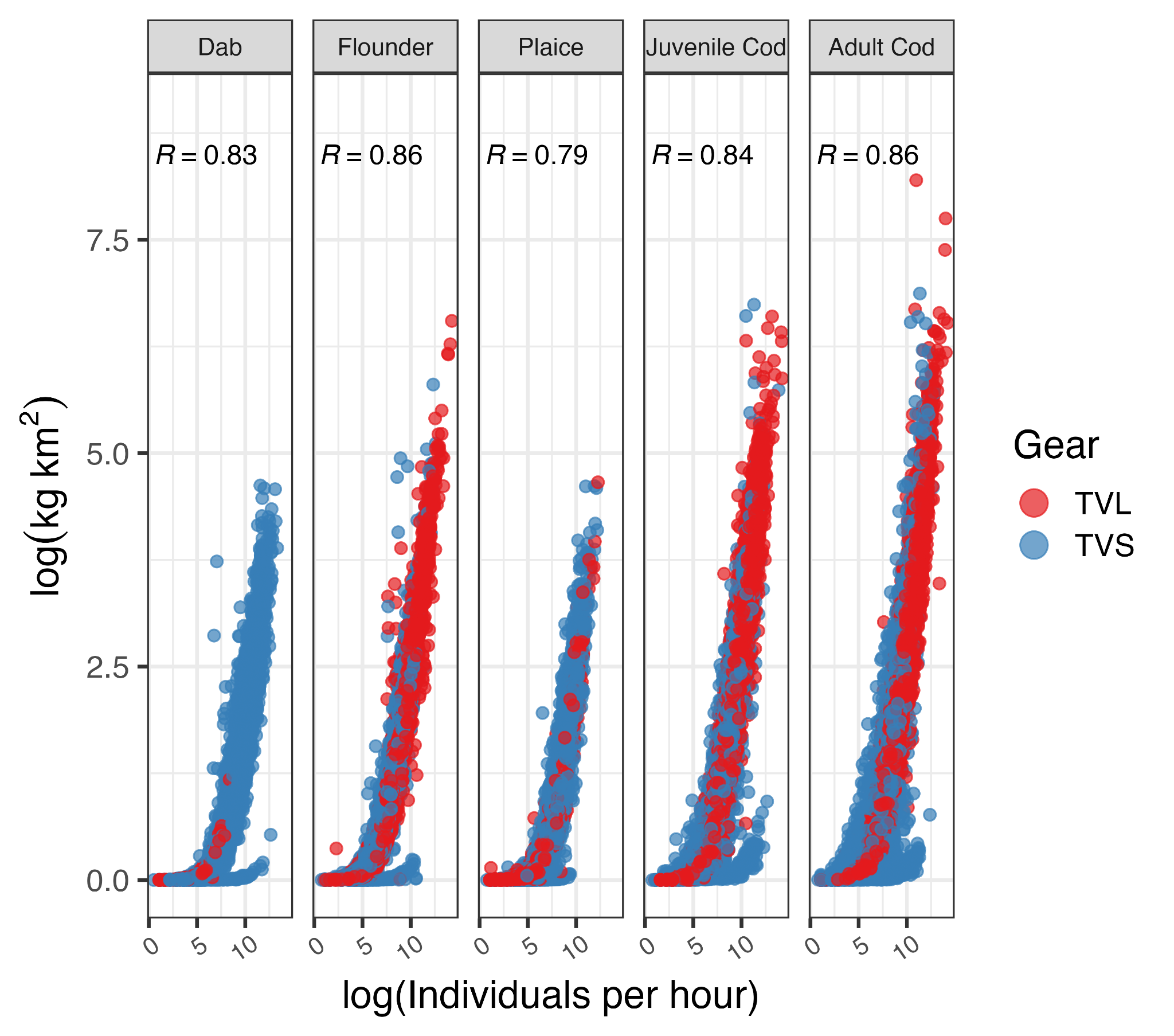


Figure S2. Biomass densities (kg km^-2^) are strongly correlated (R = Pearson’s correlation coefficient) to a conventional catch-per-unit-effort metric based on abundance counts. Trawls are colored by gear type and faceted by species.


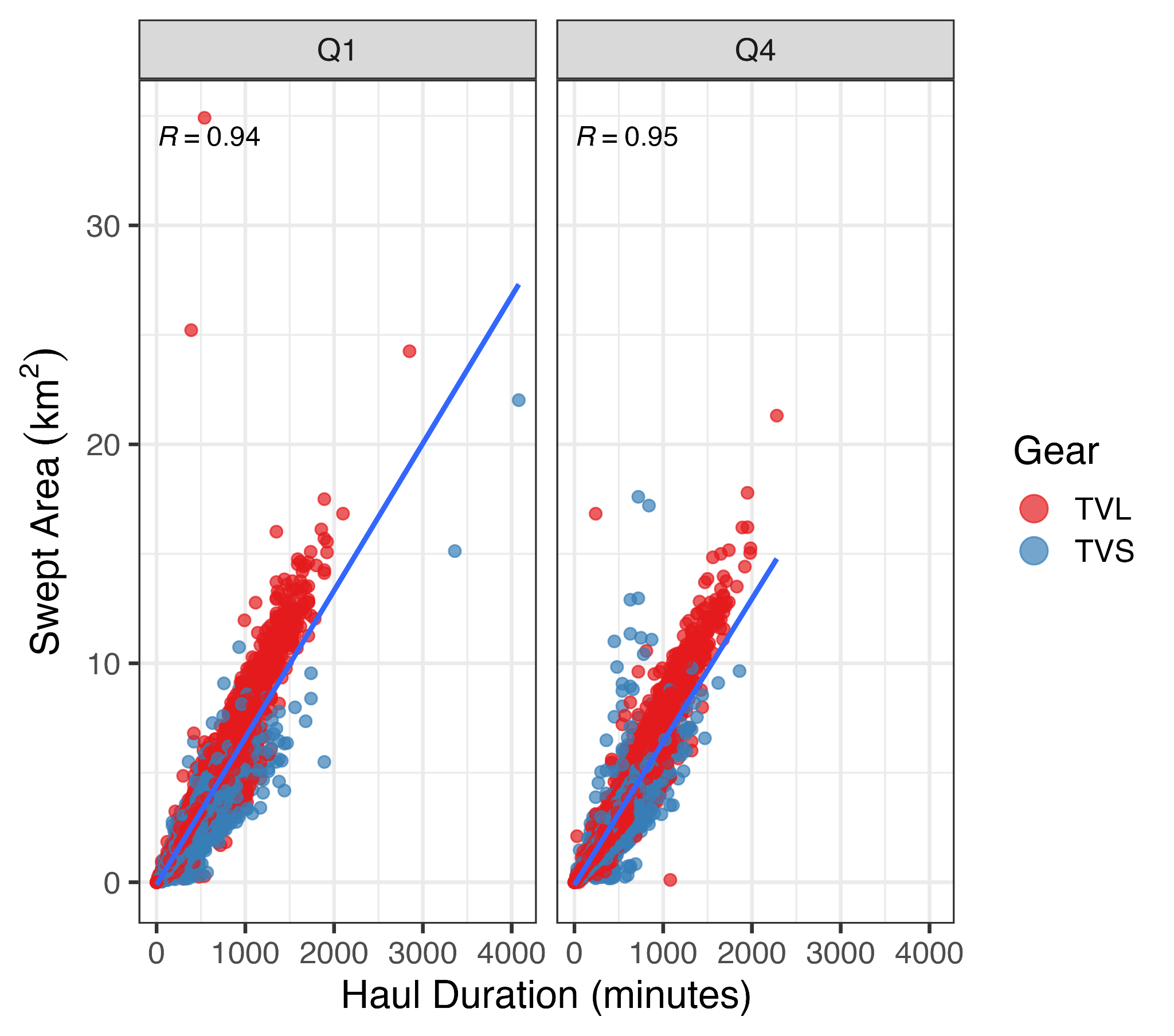


Figure S3. Swept area (km^2^) representing total covered area during trawl is strongly correlated (R = Pearson’s correlation coefficient) to haul duration (summed by station by year). Trawls are colored by gear type and faceted by quarter.


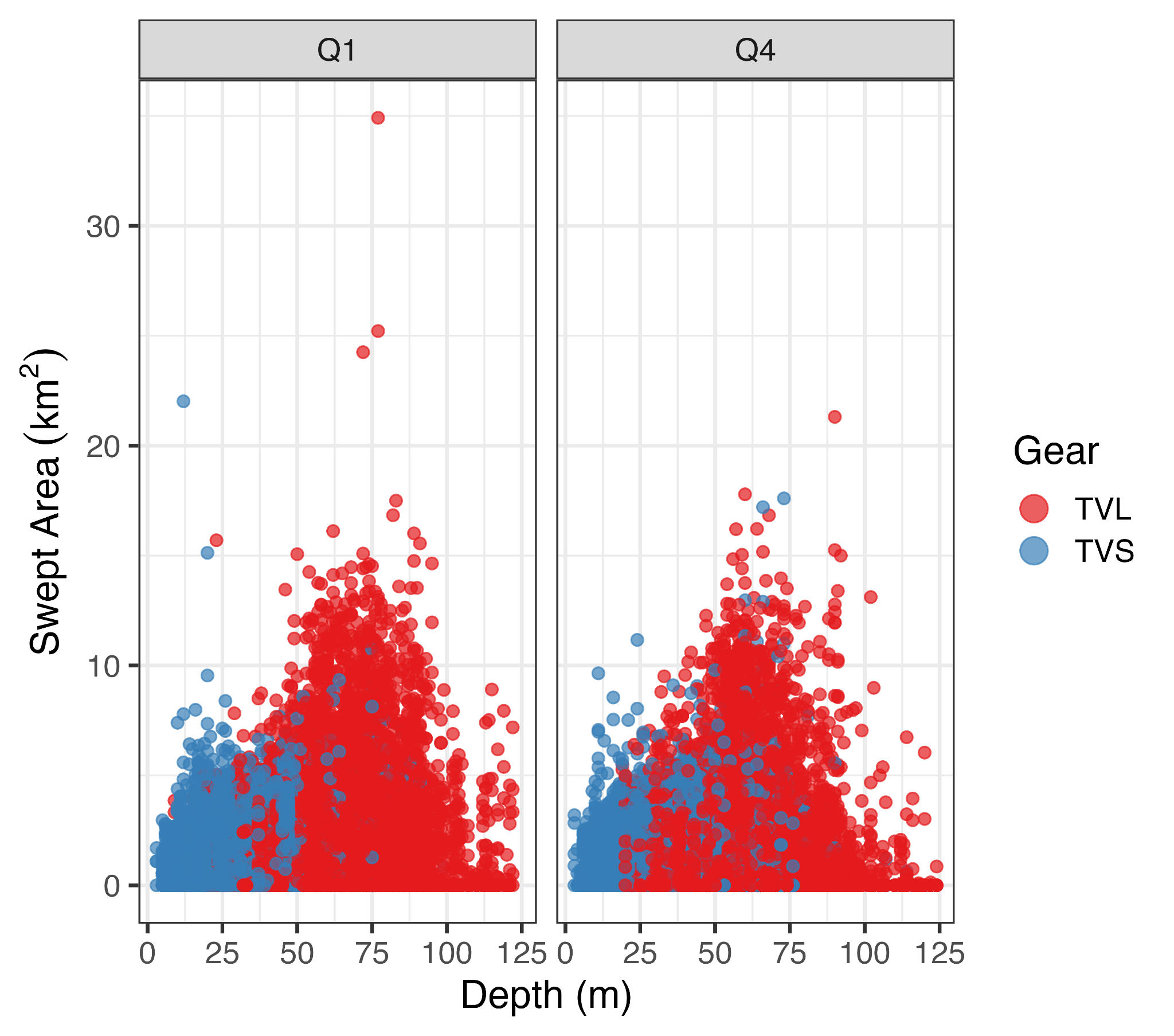


Figure S4. Swept area (km^2^) representing total covered area is distributed across depths, illustrating the partial separation of smaller TVS gear types in more shallow environments in the western Baltic Sea, and the larger TVL gear types used elsewhere in deeper regions. Trawls are colored by gear type and faceted by quarters.


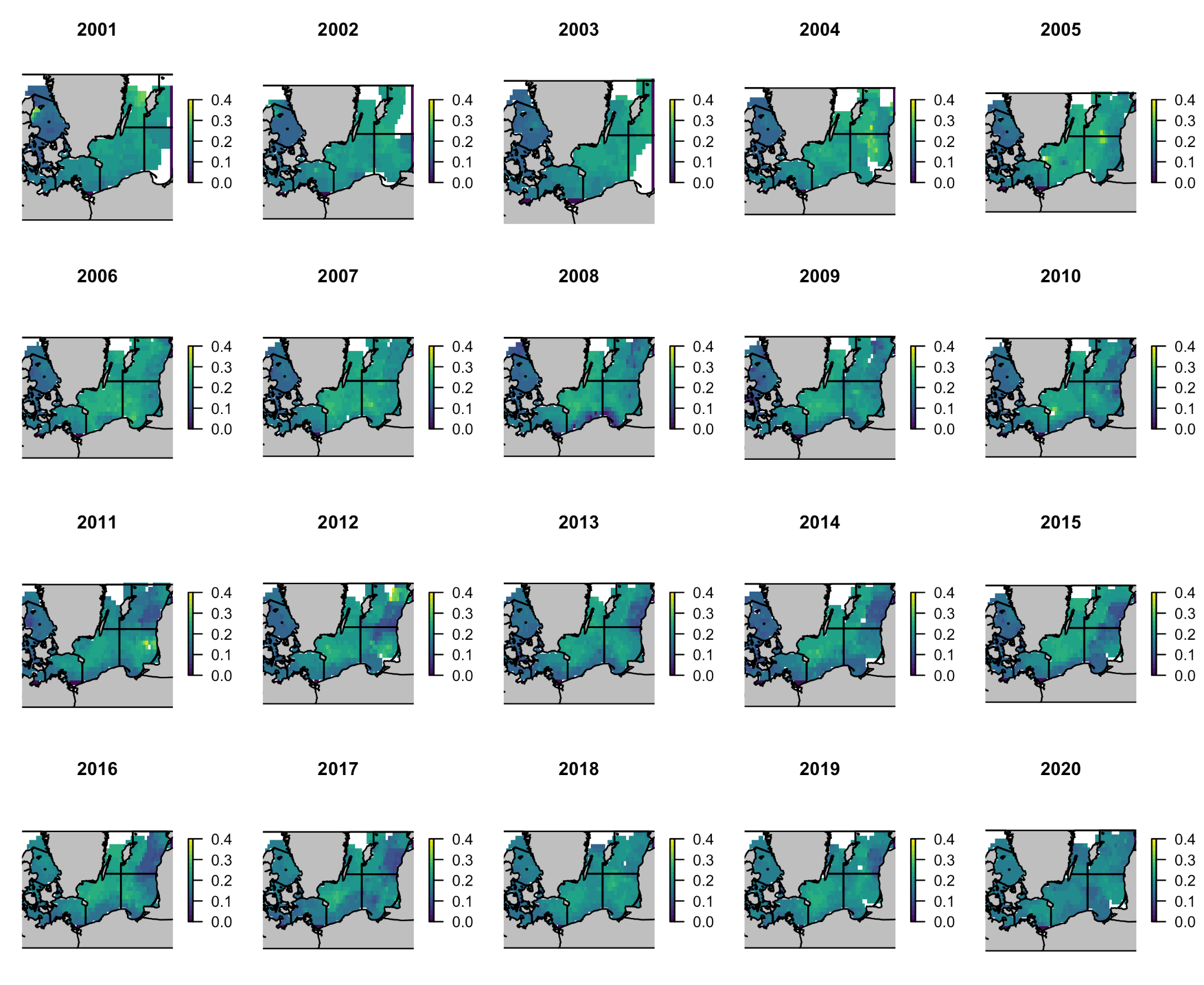


Figure S5. Door-based swept area (km^2^) corresponding to an estimate of trawl effort described in Berg *et al.* (2019). Interpolated (0.2° × 0.2°) by inverse distance weighting constrained by maximum distance of 50 km and an inverse distance power (idp = 2) prioritizing nearby points. Swept area was relatively stable throughout the 20-year time series.

Table S1. Diagnostic checks for each model version (I, II, III) including the allowed degrees of freedom set by basis function number (k), the effective degrees of freedom (EDF) used for each term, and a p-value estimate based on the simulation in the *gam.check* function in *mgcv* (Wood*,* 2017). Significant p-values (<0.05) suggest potentially inadequate basis function number for an individual smoother and motivated further checks of model residuals in Figures S8-S9.

| Smooth terms | # of basis functions (k) | EDF | F-statistic | p-value |
| --- | --- | --- | --- | --- |
| **Model-I**  ti(lon, lat, year)  Dab  Flounder  Plaice  (Juvenile) Cod  (Adult) Cod  te(year, depth)  Dab  Flounder  Plaice  (Juvenile) Cod  (Adult) Cod | 124  124  124  124  124  24  24  24  24  24 | 66.8  77.2  89  83.3  79.7  14.5  17.5  20.7  17.7  16.5 | 66.8  16.5  62.8  31.9  34.6  11.2  7.9  18.8  31.4  22.2 | <0.001  <0.001  <0.001  <0.001  <0.001  <0.005  <0.005  <0.015  <0.001  <0.001 |
| **Model II**  ti(lon, lat, year)  Dab  Flounder  Plaice  (Juvenile) Cod  (Adult) Cod  s(depth, species)  s(temperature, species)  s(oxygen, species)  s(salinity, species) | 124  124  124  124  124  225  225  225  50 | 64.3  80.9  90.4  88.6  84.9  63.9  28.1  59.4  36.7 | 35.5  21.5  48.5  26.1  29.4  5.9  1.9  4.6  11.3 | <0.001  <0.001  <0.001  <0.001  <0.001  0.3  0.04  0.005  <0.001 |
| **Model III**  ti(lon, lat, year)  Dab  Flounder  Plaice  (Juvenile) Cod  (Adult) Cod  s(depth, species)  Winter  Autumn  s(temperature, species)  Winter  Autumn  s(oxygen, species)  Winter  Autumn  s(salinity, species)  Winter  Autumn | 124  124  124  124  124  50  50  50  50  50  50  50  50 | 64.7  83.5  90.9  89.6  87.1  29.7  43.6  12.2  11.4  34.1  26.1  35.4  30.7 | 36.6  22.9  50.6  27.3  30.9  20.8  29.3  0.9  1.2  9.2  9.9  7.1  7.5 | <0.001  <0.001  <0.001  <0.001  <0.001  0.8  0.8  0.02  0.03  <0.001  <0.001  0.1  0.09 |


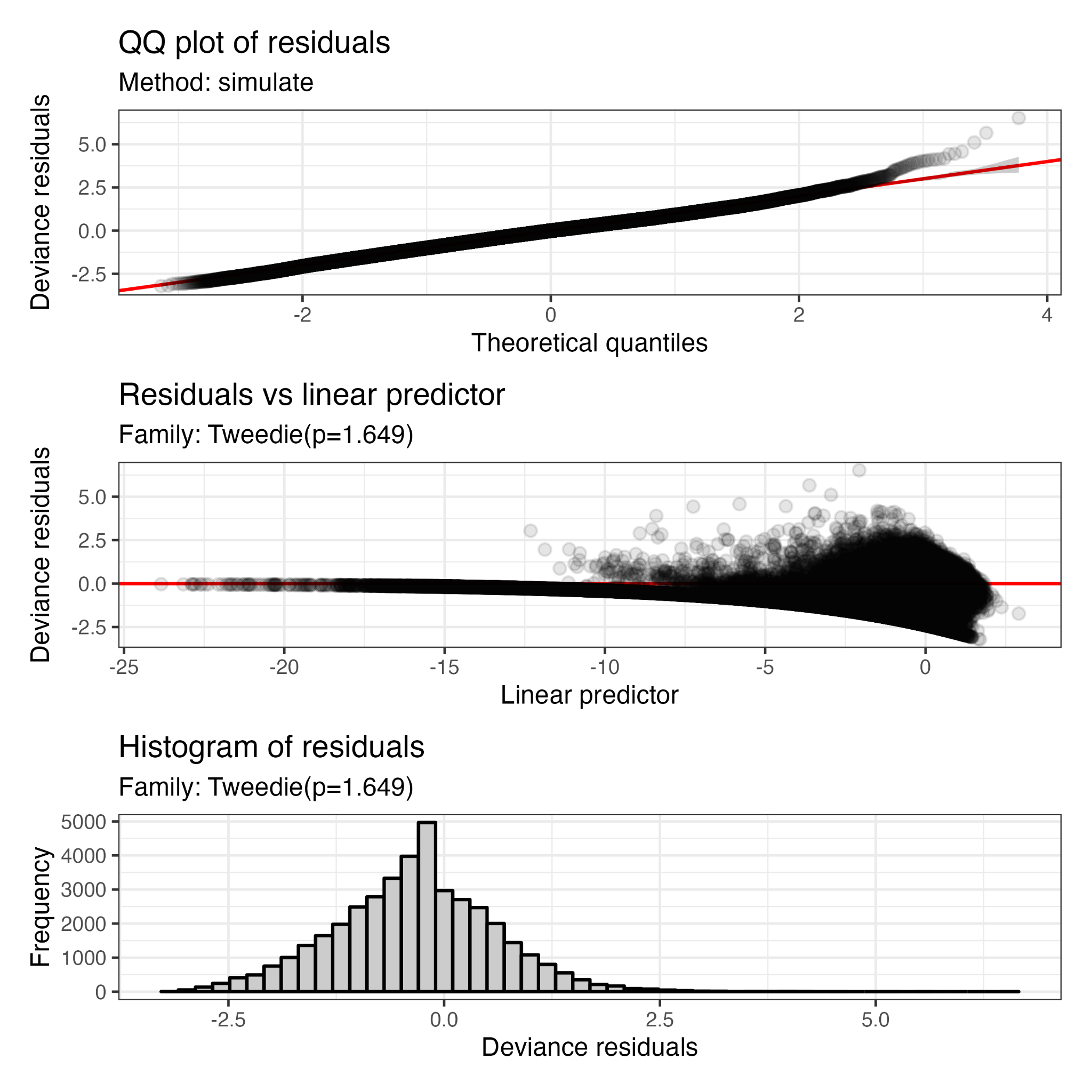


Figure S6. Diagnostic appraisal of HGAM version-III simulating QQ plots including normal standard errors, comparing the residuals to linear predictor where the zeros are the lower bound of deviance in the residuals, and finally a histogram of the HGAM residuals. An additional diagnostic plot comparing the response variable versus fitted values is shown below in Figure S7 including species-specific differences across 10-fold cross validation.


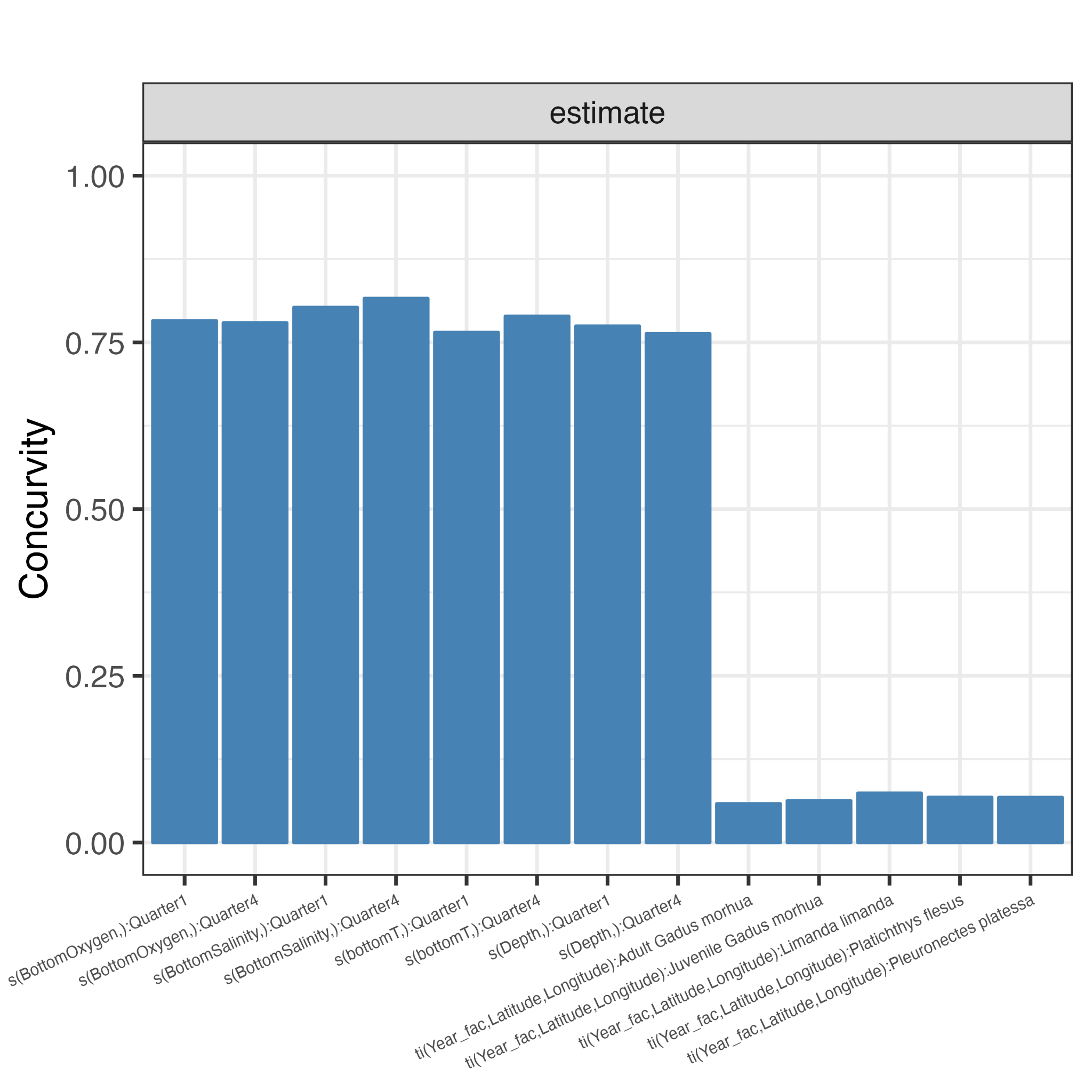


Figure S7. HGAM version-III model concurvity for smooth terms (Simpson, 2018), where a value of 1 indicates complete non identifiability (analogous to maximal collinearity). No consensus threshold exists for excluding terms based on concurvity, but we note that all values for all models are beneath 0.8 (e.g., Leonardi *et al.* 2022).


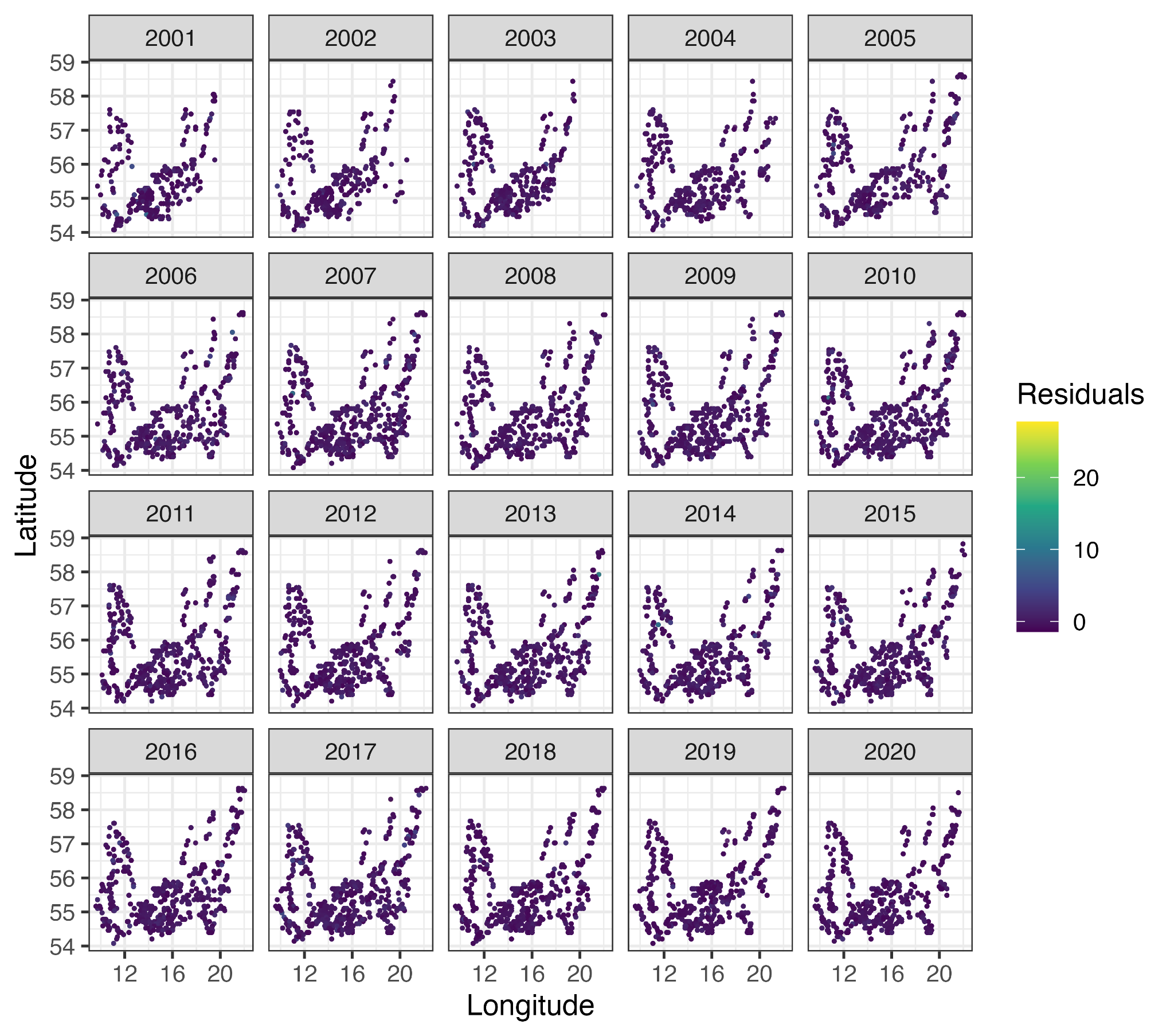


Figure S8. Model residuals (HGAM version-III) plotted geographically (Longitude versus Latitude) and by year, with residual magnitude filling point color. No clear model residual patterns against space or time are evident.


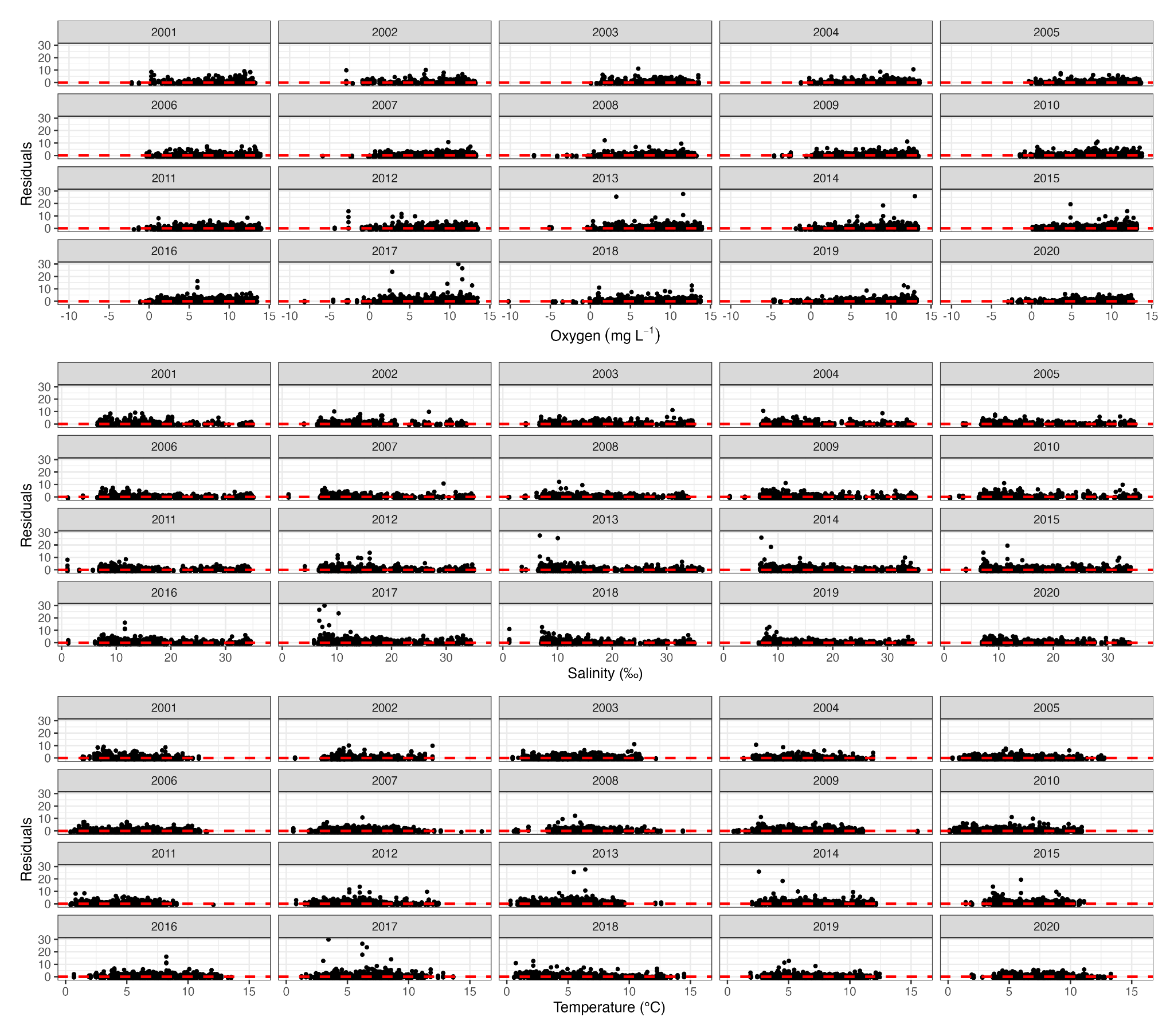


Figure S9. Model residuals (HGAM version-III) plotted against environmental covariates and by year. The red dotted line is set at zero (y = 0) and altogether no dependencies between model residuals and abiotic covariates are apparent.


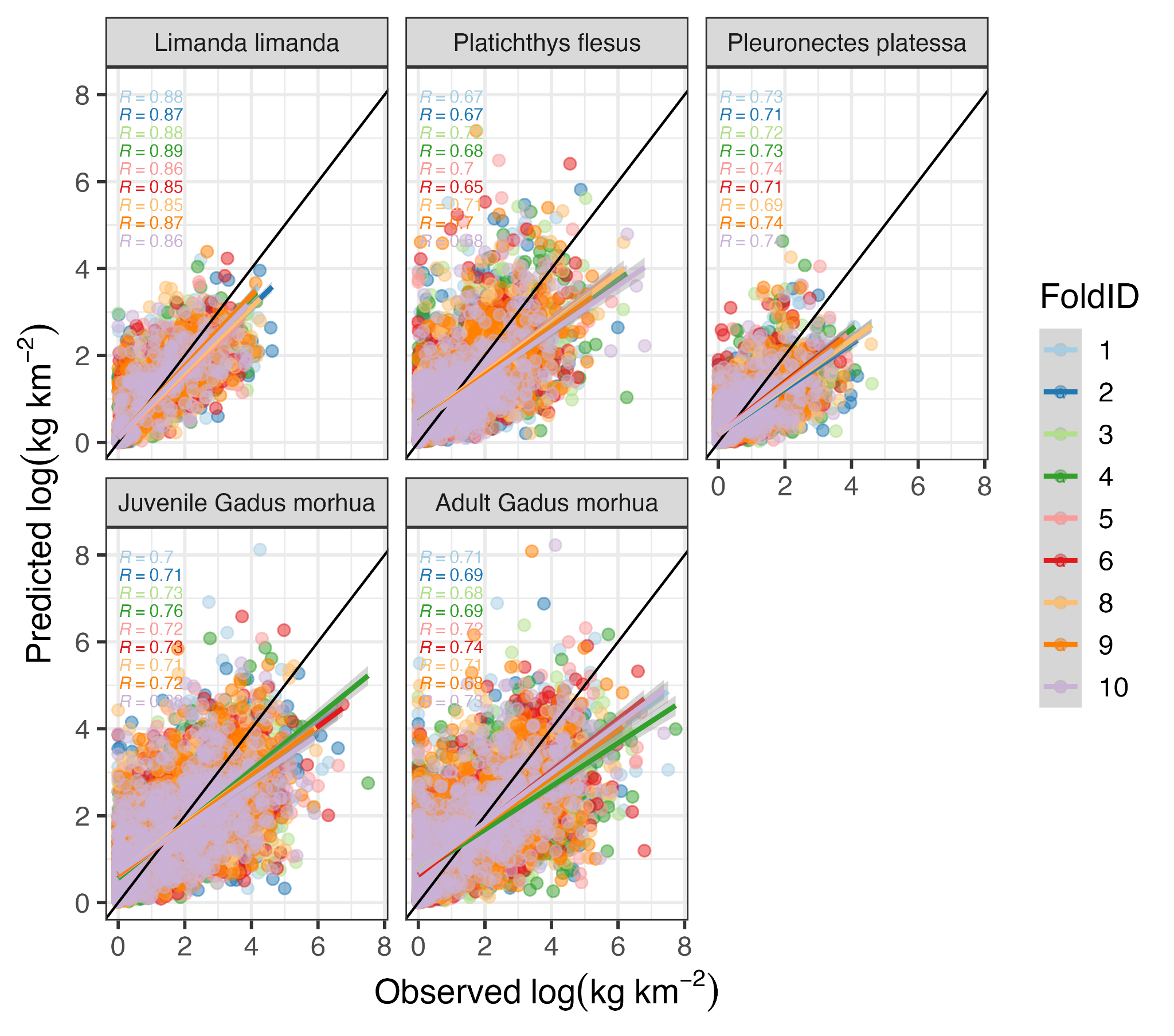


Figure S10. Diagnostic appraisal of HGAM version-III for predictive performance in 10-fold cross validation, comparing observed versus fitted biomass on the natural log scale. Points and linear corrections are colored by k-fold, with Pearson’s correlation coefficient (R) recorded in each panel for each k-fold. The 9th fold model run failed to converge and was excluded. The solid black line represents a perfect linear relationship for comparison. The average linear correlations (with standard deviations) represent our measure of discrimination recorded in Table S2. The two additional measures, precision and accuracy, also derive from these diagnostics with point cloud dispersion capturing precision and the absolute error in prediction values capturing accuracy (Table S2).

Table S2. HGAM performance by species group and model version (I, II, III) against regression metrics for predictive performance (discrimination, precision, accuracy) and parsimony (AIC). The optimal regression metric values (O_v_) are as follows: Discrimination (O_v_=1), Precision (O_v_=1), Accuracy (O_v_=0).

| Model | Group | Discrimination  (Correlation) | Precision  (Dispersion) | Accuracy  (MAE) | Deviance explained | AIC |
| --- | --- | --- | --- | --- | --- | --- |
| Version-I (Geographic) | Dab  Flounder  Plaice  (Juvenile) Cod  (Adult) Cod | 0.83 ± 0.016  0.52 ± 0.03  0.68 ± 0.028  0.56 ± 0.022  0.68 ± 0.016 | 0.75 ± 0.042  0.31 ± 0.019  0.53 ± 0.064  0.41 ± 0.029  0.49 ± 0.027 | 0.23 ± 0.015  0.70 ± 0.02  0.30 ± 0.012  0.88 ± 0.022  0.85 ± 0.024 | 61.0% | 54218 |
| Version-II  (+ Abiotic) | Dab  Flounder  Plaice  (Juvenile) Cod  (Adult) Cod | 0.86 ± 0.011  0.59 ± 0.021  0.71 ± 0.028  0.70 ± 0.017  0.70 ± 0.013 | 0.81 ± 0.049  0.48 ± 0.031  0.60 ± 0.041  0.59 ± 0.030  0.56 ± 0.018 | 0.20 ± 0.014  0.65 ± 0.013  0.28 ± 0.010  0.75 ± 0.025  0.78 ± 0.019 | 62.1% | 53107 |
| Version-III  (+ Seasonal Abiotic) | Dab  Flounder  Plaice  (Juvenile) Cod  (Adult) Cod | 0.87 ± 0.013  0.68 ± 0.026  0.74 ± 0.013  0.73 ± 0.0224  0.72 ± 0.019 | 0.83 ± 0.056  0.67 ± 0.015  0.63 ± 0.055  0.62 ± 0.035  0.61 ± 0.032 | 0.20 ± 0.010  0.56 ± 0.013  0.28 ± 0.013  0.74 ± 0.024  0.78 ± 0.021 | 65.2% | 50211 |

**
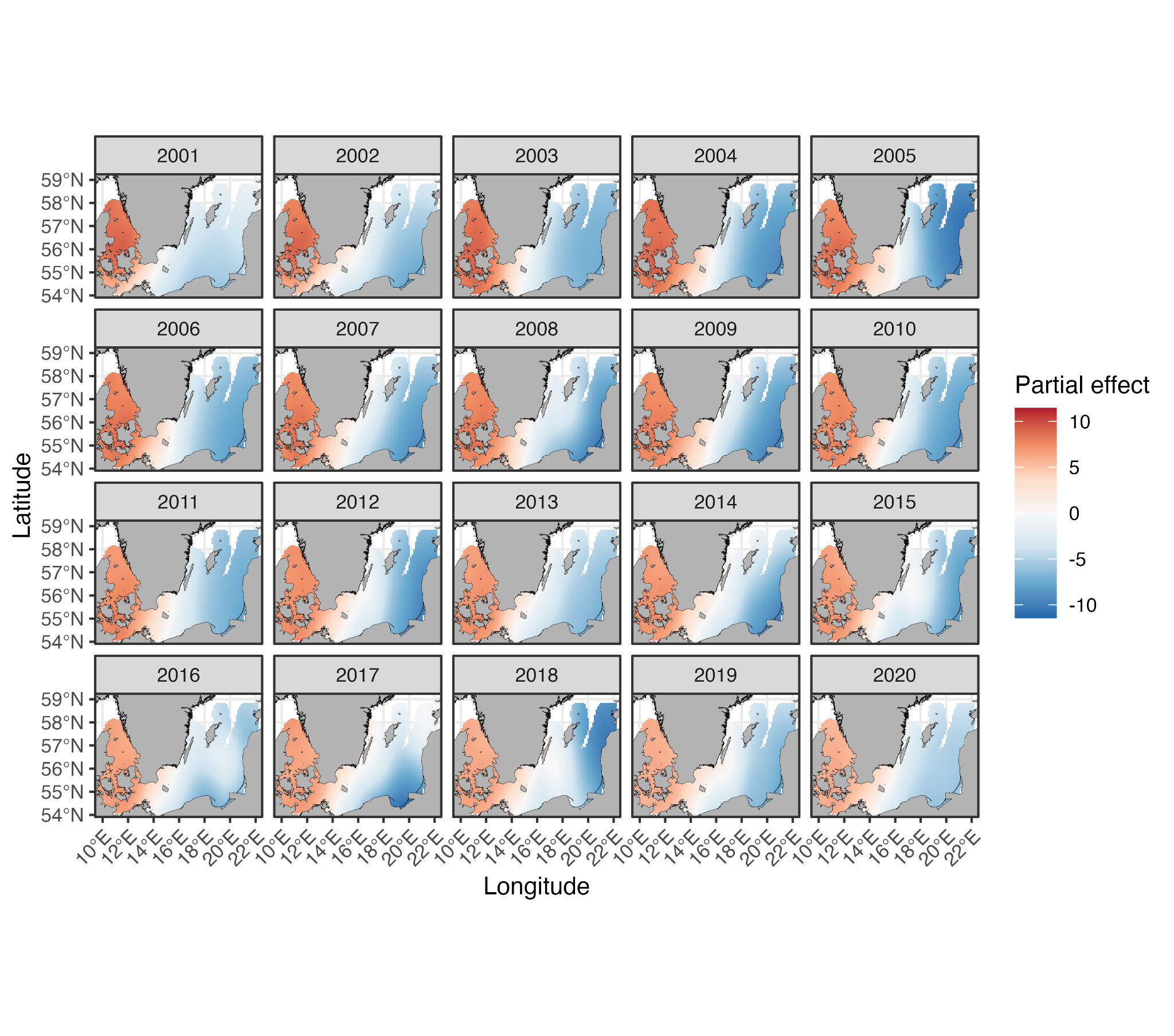
**

Figure S11. Common dab (*Limanda limanda*) spatiotemporal partial dependence (2-d smooth; Simpson, 2018) for tensor smooth product (te(lon, lat, year, by = species)) in HGAM version-III. The 2-D smooth maps are cropped by the 0.5° buffer polygon to avoid extrapolation into unsampled areas.

**
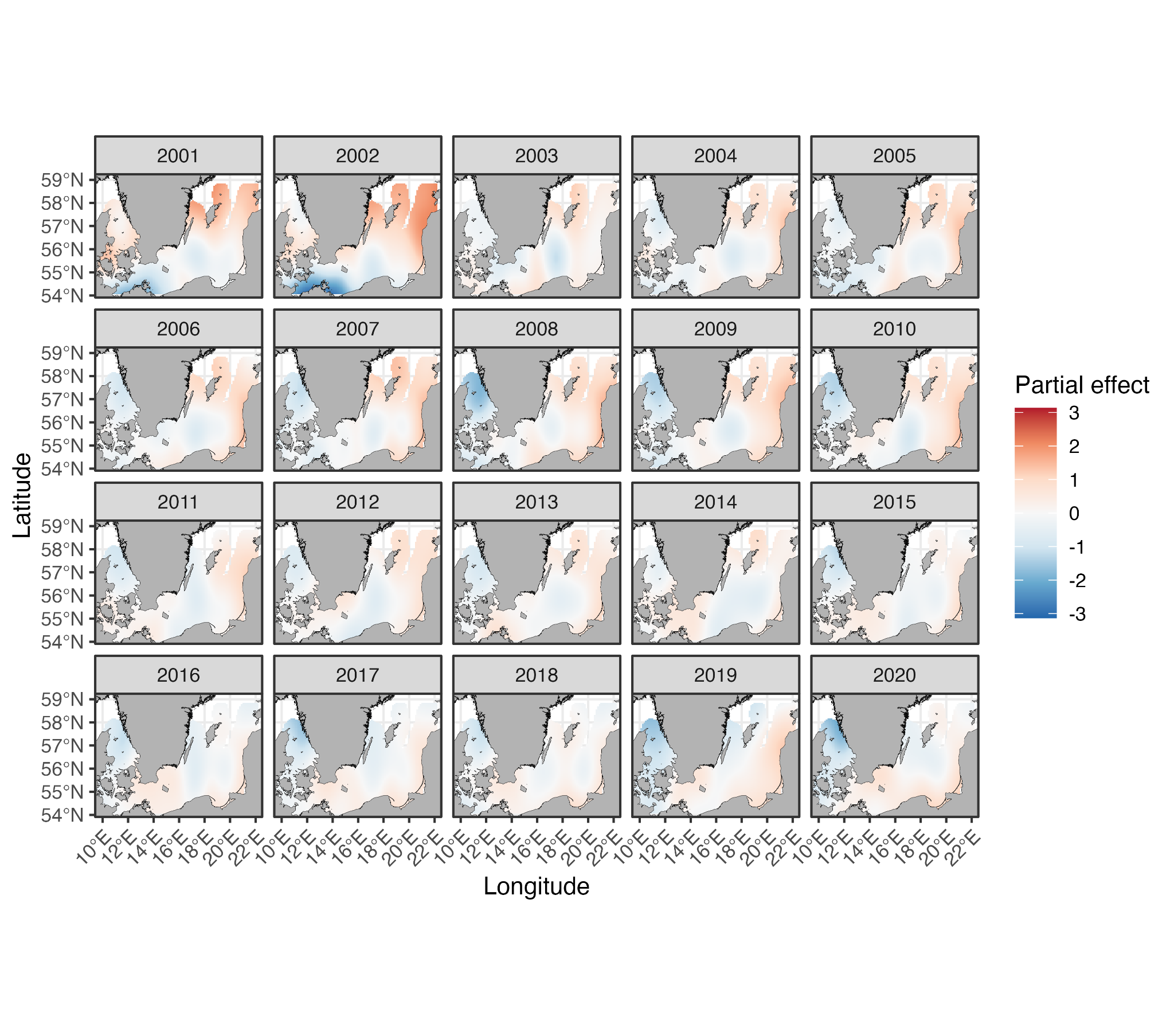
**

Figure S12. European flounder (*Platichthys flesus*) spatiotemporal partial dependence (2-d smooth; Simpson, 2018) for tensor smooth product (te(lon, lat, year, by = species)) in HGAM version-III. The 2-D smooth maps are cropped by the 0.5° buffer polygon to avoid extrapolation into unsampled areas.

**
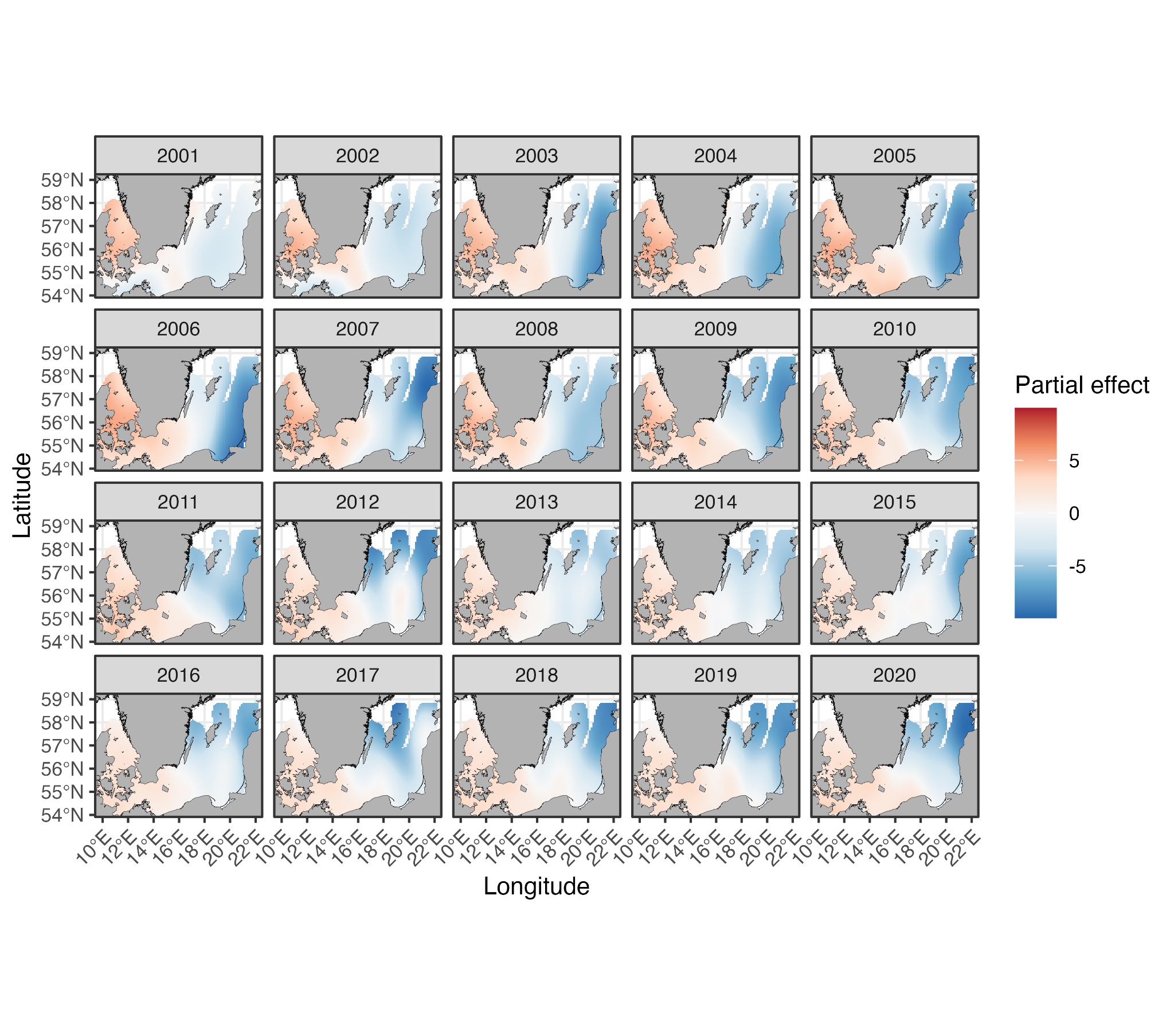
**

Figure S13. European plaice (*Pleuronectes platessa*) spatiotemporal partial dependence (2-d smooth; Simpson, 2018) for tensor smooth product (te(lon, lat, year, by = species)) in HGAM version-III. The 2-D smooth maps are cropped by the 0.5° buffer polygon to avoid extrapolation into unsampled areas.

**
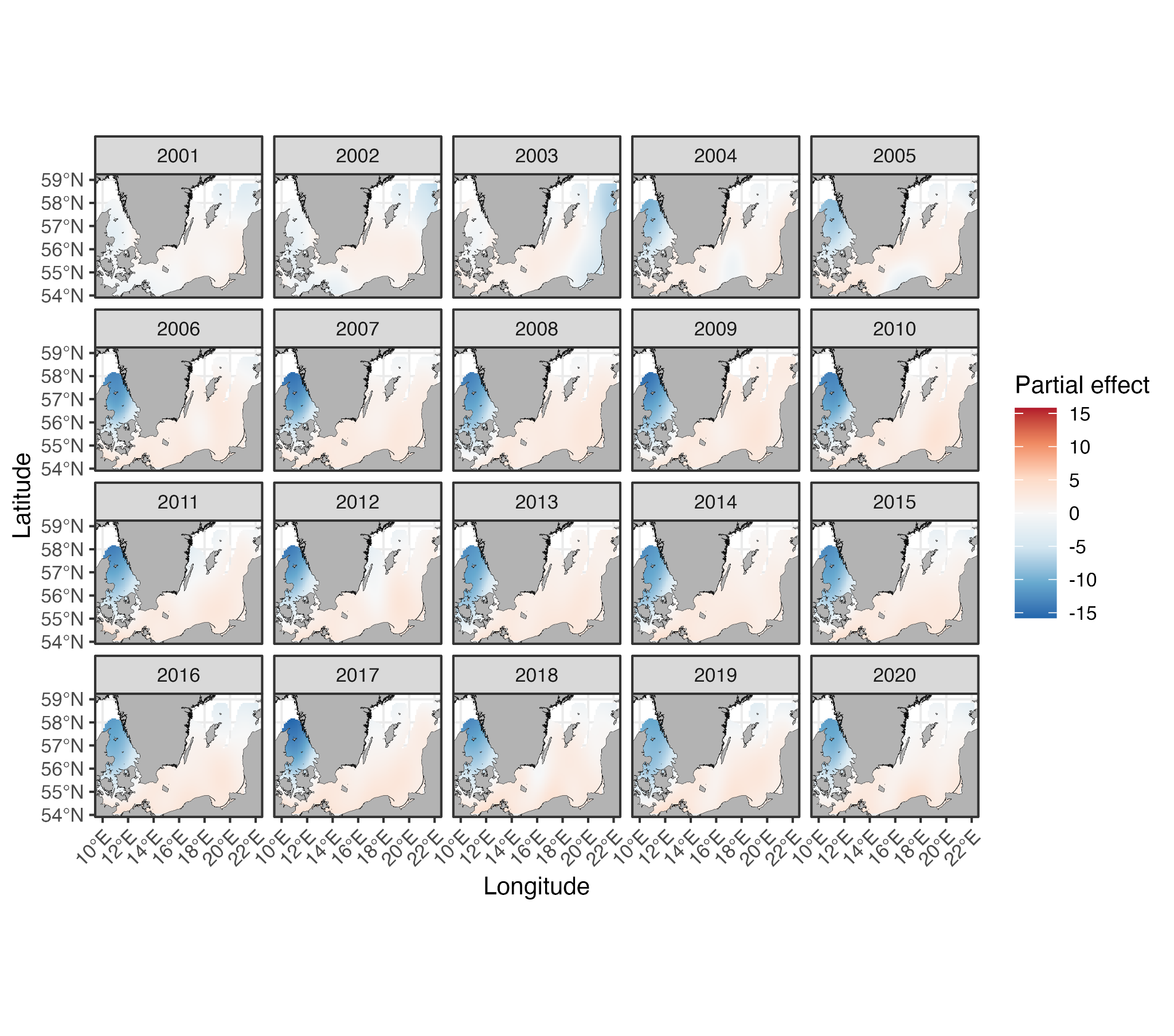
**

Figure S14. Juvenile atlantic cod (*Gadus morhua*, <35 cm) spatiotemporal partial dependence (2-d smooth; Simpson, 2018) for tensor smooth product (te(lon, lat, year, by = species)) in HGAM version-III. The 2-D smooth maps are cropped by the 0.5° buffer polygon to avoid extrapolation into unsampled areas.

**
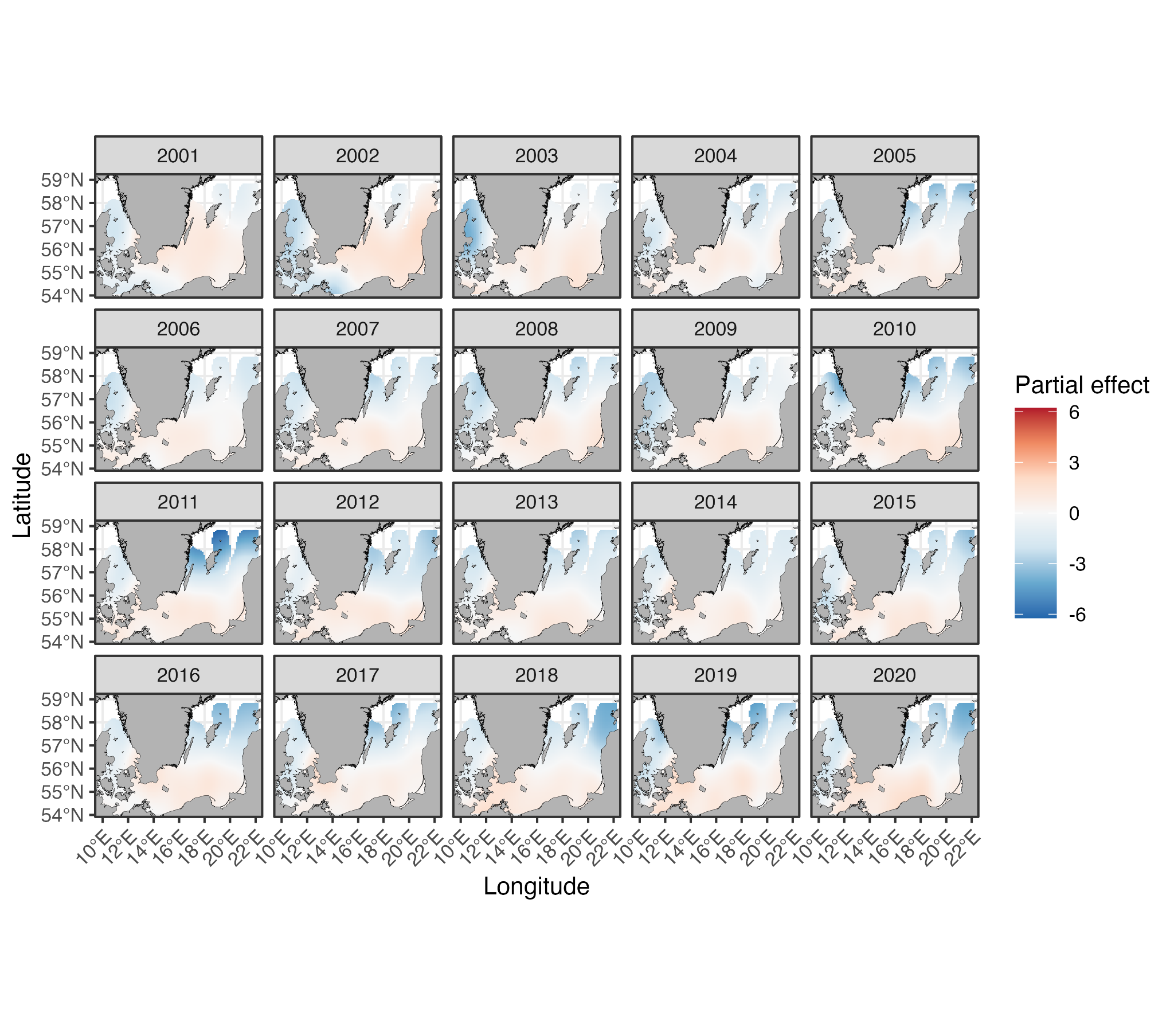
**

Figure S15. Adult atlantic cod (*Gadus morhua*, ≥35 cm) spatiotemporal partial dependence (2-d smooth; Simpson, 2018) for tensor smooth product (te(lon, lat, year, by = species)) in HGAM version-III. The 2-D smooth maps are cropped by the 0.5° buffer polygon to avoid extrapolation into unsampled areas.

**
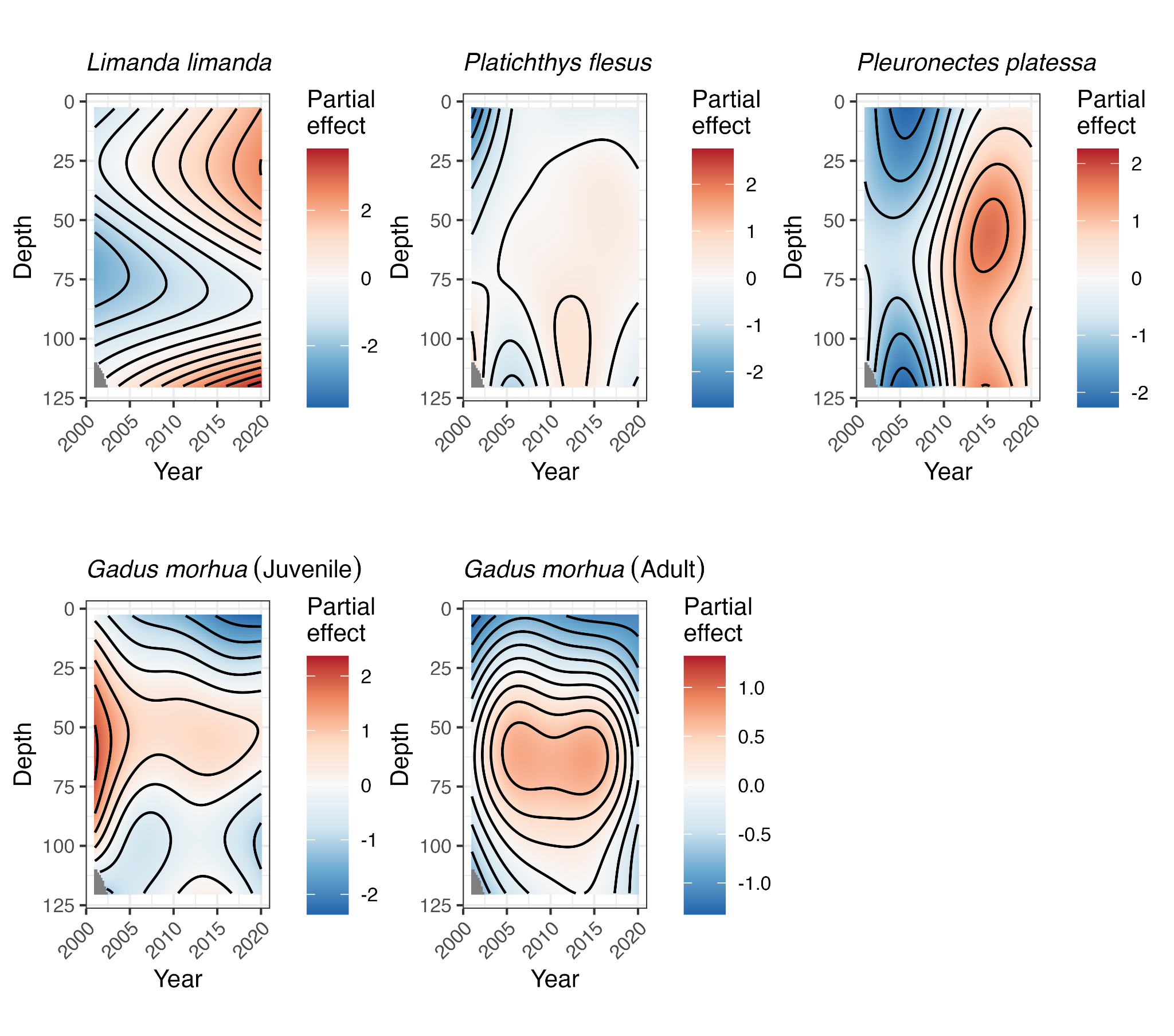
**

Figure S16. Spatiotemporal partial dependence (2-d smooth; Simpson, 2018) for tensor smooth product (te(depth, year, by = species)) in HGAM version-I. Depth is measured in metres. This term was simplified to s(depth, species) in subsequent model versions (II and III) to avoid high concurvity and did not impact model predictive performance or abiotic partial dependence curves. The greyed out corner at deeper depths (110-125 m) from 2001-2002 inclusively represents a distance (default dist = 0.01) constraint in the smooth estimation to avoid spurious extrapolation.


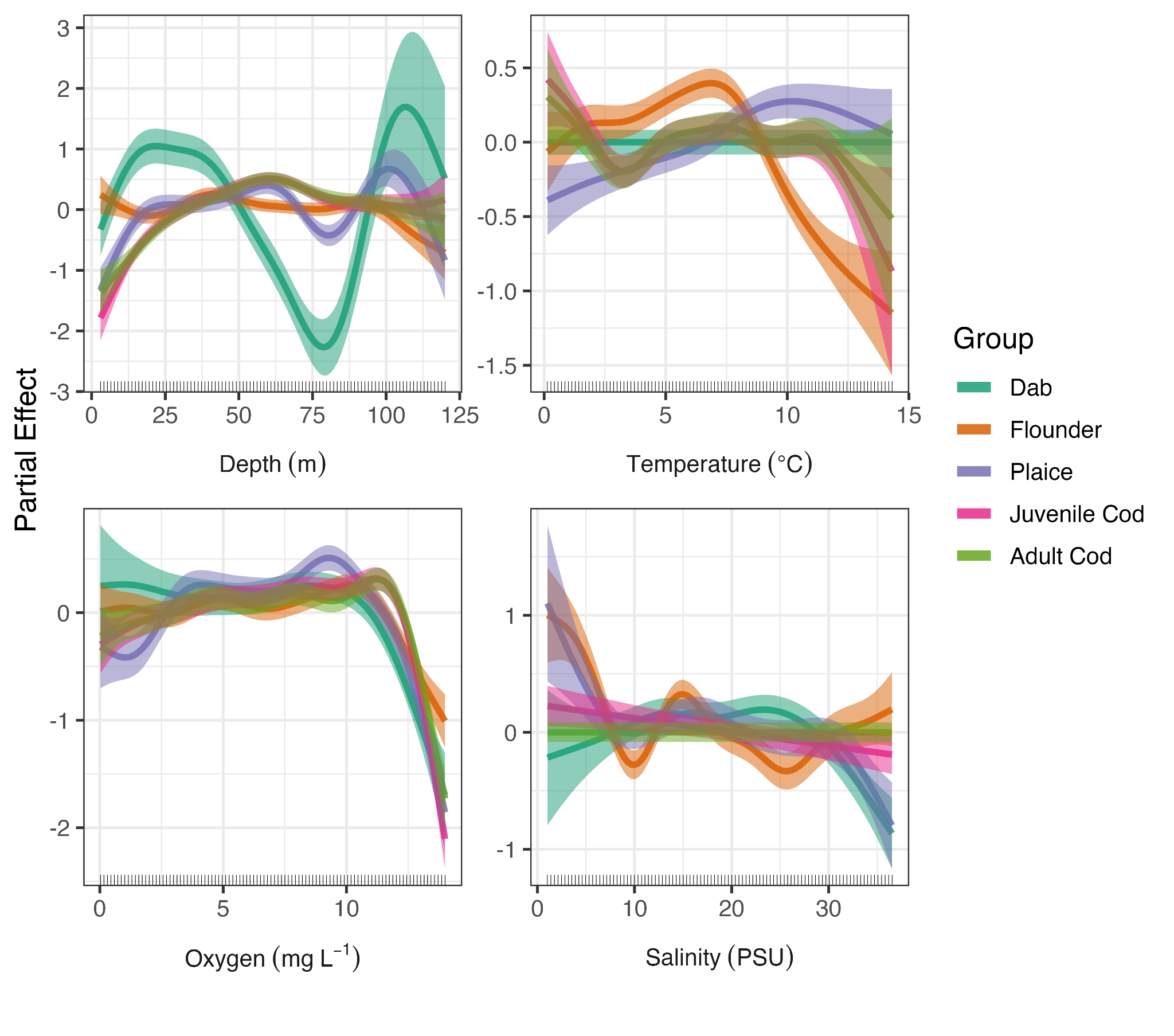


Figure S17. Spatiotemporal partial dependence (Simpson, 2018) for factor smooths of depth and abiotic covariates in HGAM version-II, ignoring seasonality. This model version outperformed the geographic-only model (HGAM version-I) but was inferior to HGAM version-III (seasonal covariates) based on predictive performance and AIC. 95% confidence intervals are shown for each species partial effect, and x-axis rugs mark the observed data coverage on the x-axis for each covariate.

**
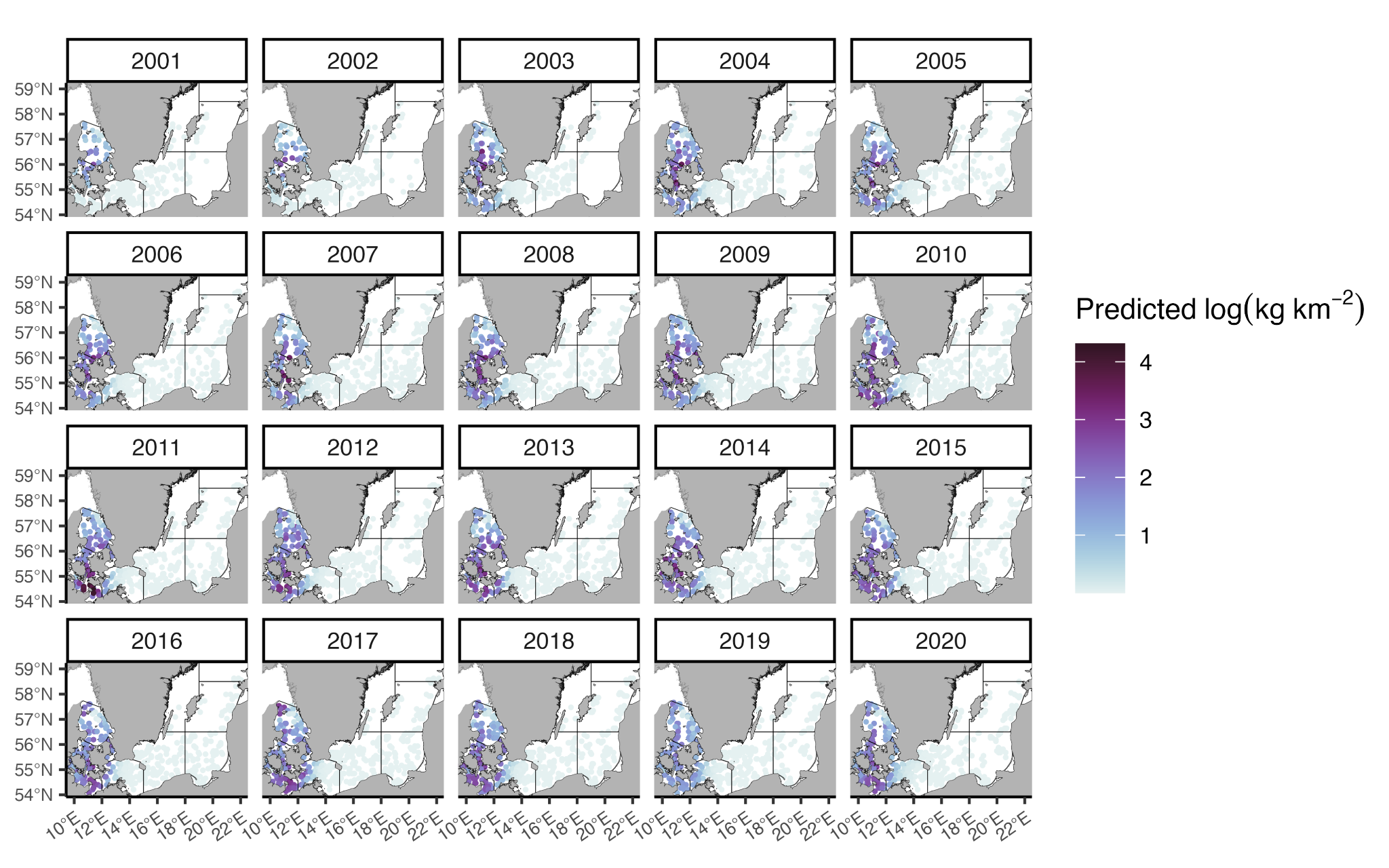
**

Figure S18. Common dab (*Limanda limanda*) biomass predictions for HGAM version-III, expressed as log(kg km^-2^), for the full time series (2001-2020).

**
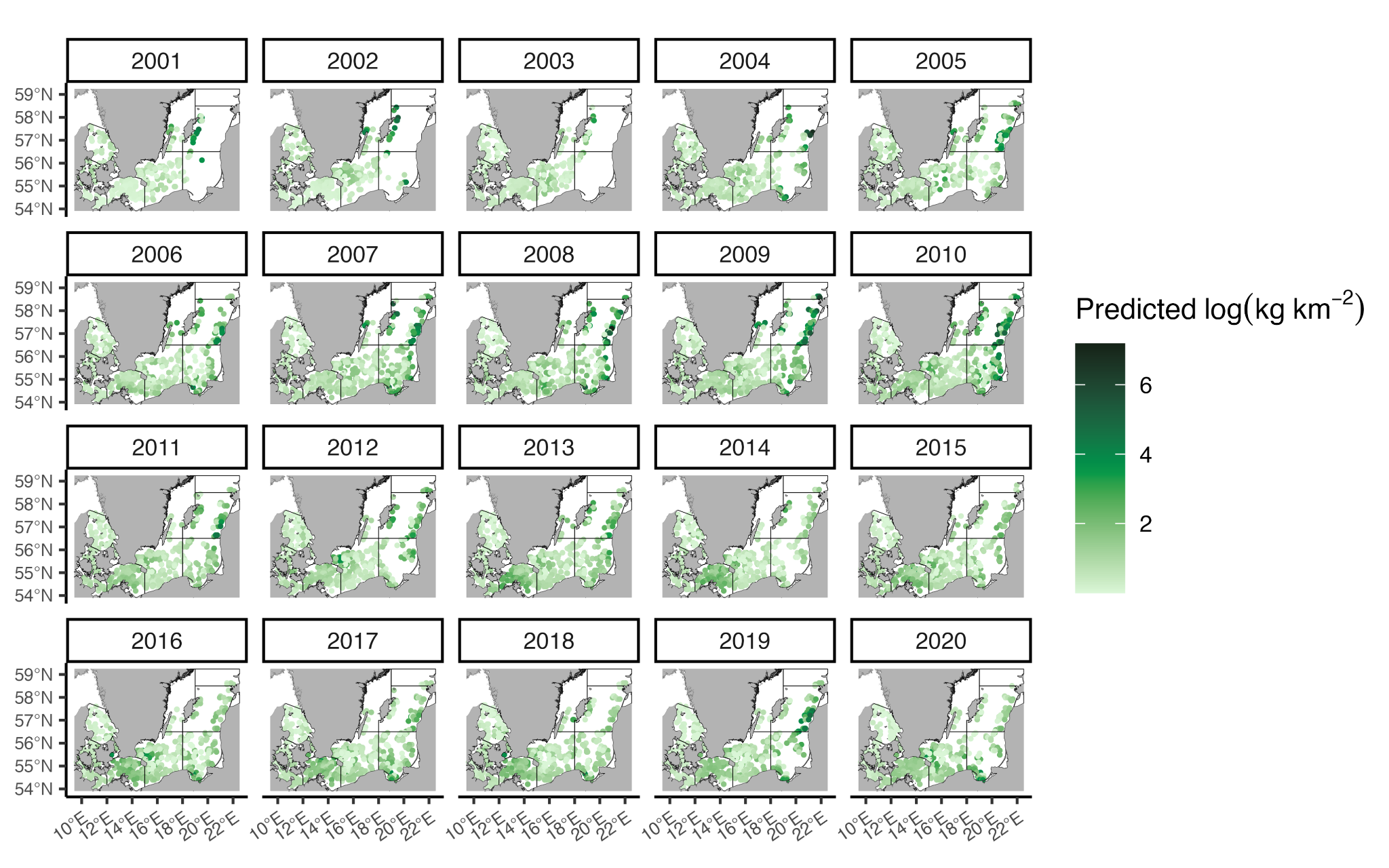
**

Figure S19. European flounder (*Platichthys flesus*) biomass predictions HGAM version-III, expressed as log(kg km^-2^), for the full time series (2001-2020).

**
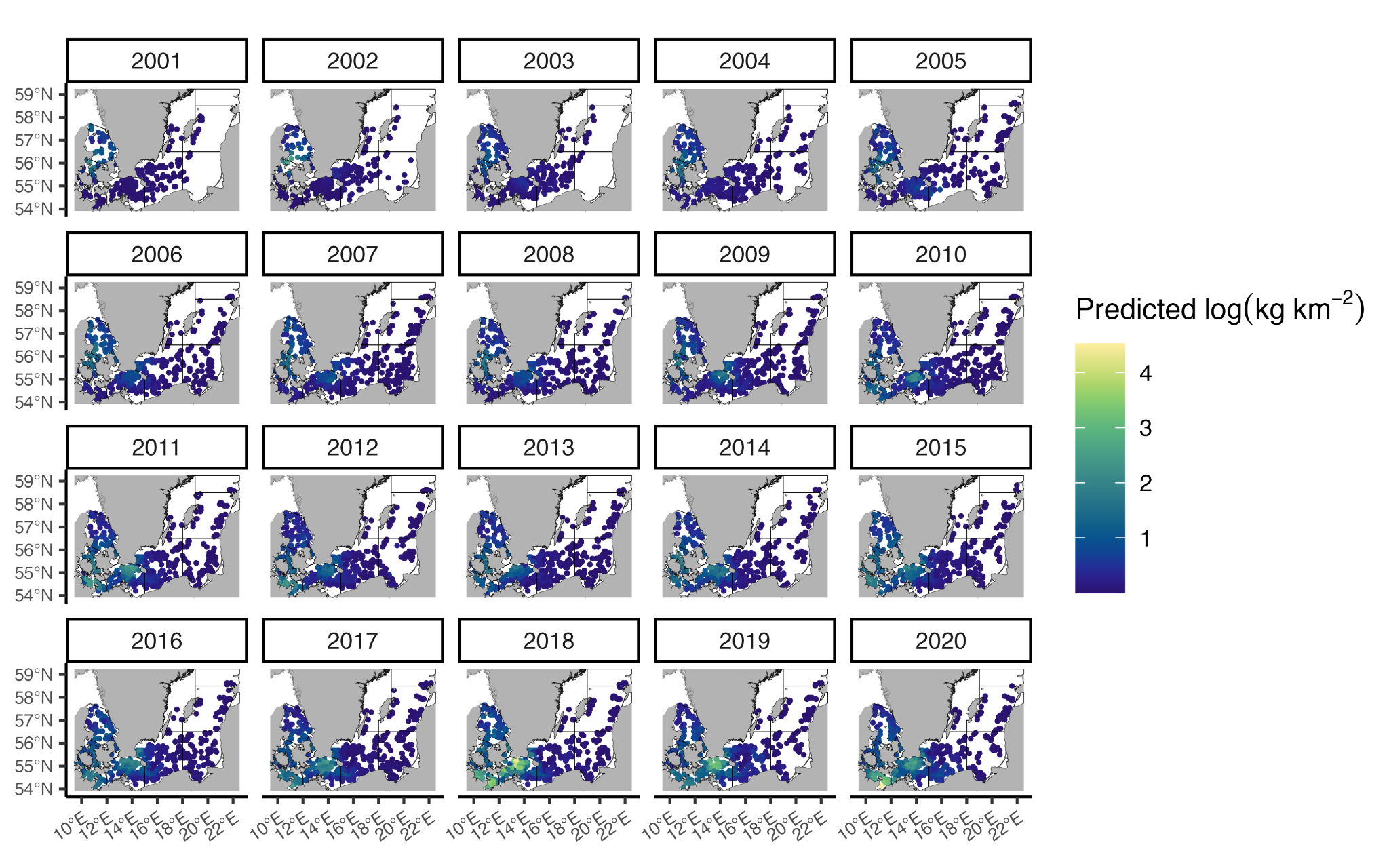
**

Figure S20. European plaice (*Pleuronectes platessa*) biomass predictions HGAM version-III, expressed as log(kg km^-2^), for the full time series (2001-2020).

**
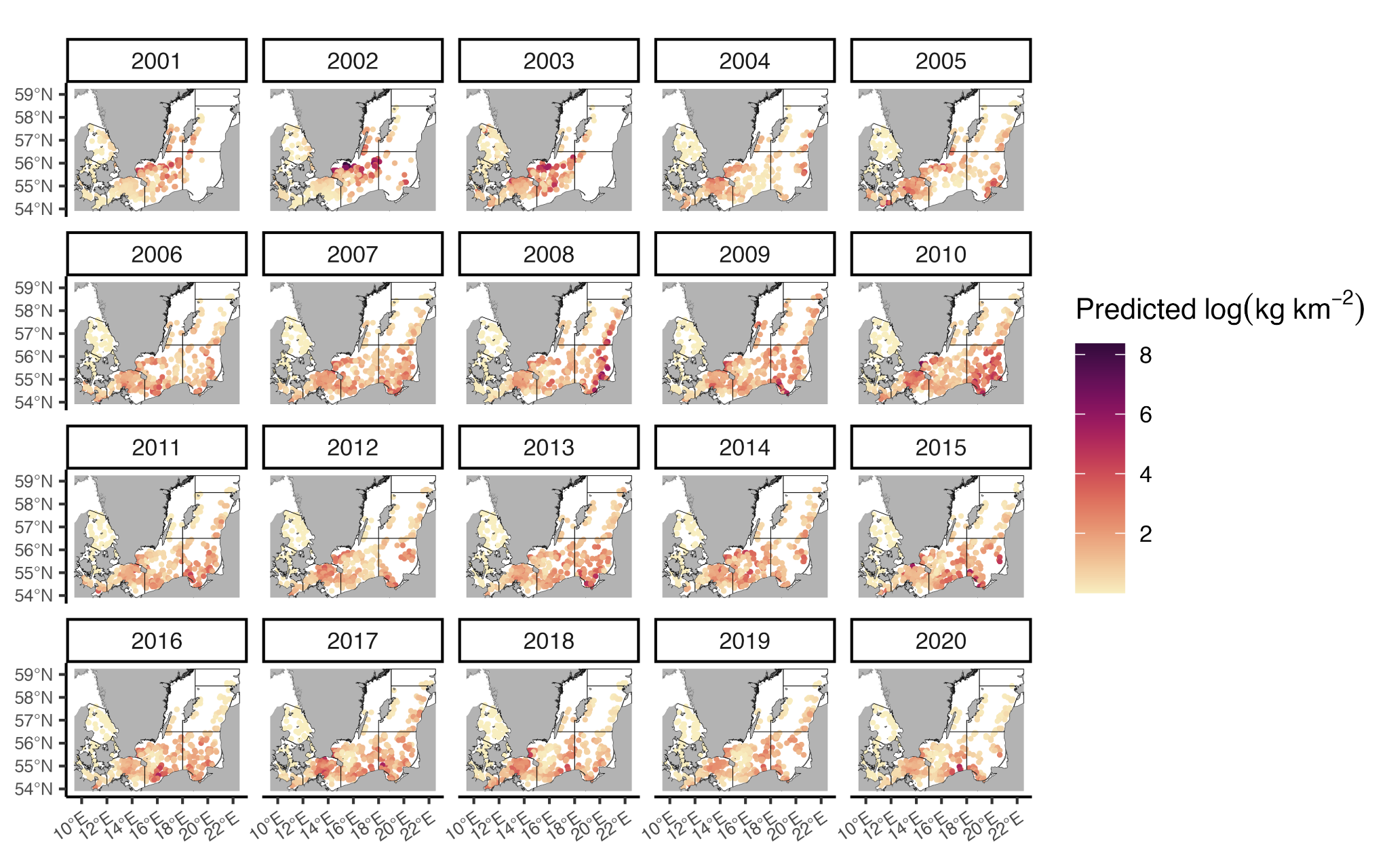
**

Figure S21. Juvenile Atlantic cod (*Gadus morhua*) biomass predictions HGAM version-III, expressed as log(kg km^-2^), for the full time series (2001-2020).

**
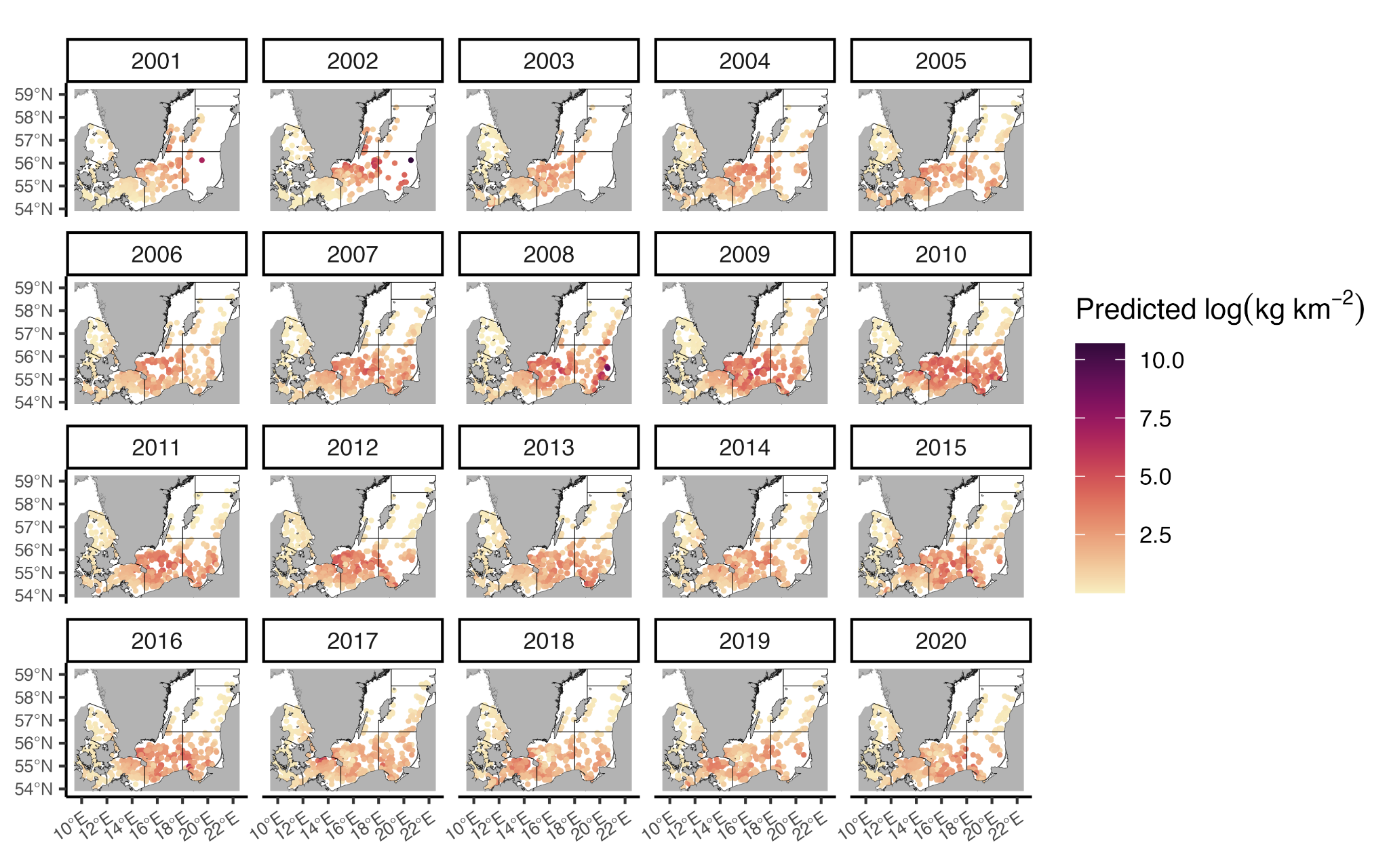
**

Figure S22. Adult Atlantic cod (*Gadus morhua*) biomass predictions HGAM version-III, expressed as log(kg km^-2^), for the full time series (2001-2020).

**References**

Berg, C. W., Brun, M., Börjesson, P., Chaves, C., Degel, H., Lynam, C. P., Martinez, I., Schuchert, P., Soni, V., Velaso, F., Villamor, A., & Wieland, K. (2019). Workshop on Methods to develop a swept-area based effort index (WKSABI). International Council for the Exploration of the Sea (ICES). ICES Scientific Report Vol. 1 No. 3 <https://doi.org/10.17895/ices.pub.4902>

Leonardi, M., Boschin, F., Boscato, P., & Manica, A. (2022). Following the niche: The differential impact of the last glacial maximum on four European ungulates. *Communications Biology*, *5*(1), 1038.<https://doi.org/10.1038/s42003-022-03993-7>

Simpson, G.L. (2018). R Package: gratia. Ggplot-based graphics and other useful functions for GAMs fitted using Mgcv, 0.1-0 (Ggplot-based graphics and utility functions for working with GAMs fitted using the mgcv package).
