# Supplementary material for: Spatial Change of Dominant Baltic Sea Demersal Fish Across Two Decades
Source: Ecol Evol. 2025 Apr 21;15(4):e71309. doi: 10.1002/ece3.71309 (PMC12011422; doi:10.1002/ece3.71309)
Supplement: Supplementary file 2 — Appendix S2. [file ECE3-15-e71309-s001.docx]

**Appendix S2:**

**ODMAP Protocol: Following Zurrel *et al.* (2020)**

**Spatial change of dominant Baltic Sea demersal fish across two decades**

Liam MacNeil^1^(<https://orcid.org/0000-0002-4125-5240>), Frane Madiraca^2^ (<https://orcid.org/0009-0005-6482-5081>), Saskia Otto^2^, Marco Scotti^1,3^ (<https://orcid.org/0000-0002-0775-6148>)

^1^ Department of Marine Ecology, GEOMAR Helmholtz Centre for Ocean Research Kiel, Kiel, Germany

^2^ [Institute for Marine Ecosystem and Fisheries Science, University of Hamburg, Hamburg, Germany](https://www.researchgate.net/institution/University_of_Hamburg)

^3^ Institute of Biosciences and Bioresources, National Research Council of Italy, Sesto Fiorentino, Italy

Correspondence: [lmacneil@geomar.de](mailto:lmacneil@geomar.de)

**Overview**

Authorship

Contact: [lmacneil@geomar.de](mailto:lmacneil@geomar.de)

Model Objective

Mapping and interpolation

**Target output:** Spatially continuous biomass density (kg km^-2^).

Focal Taxa

Atlantic Cod (*Gadus morhua*)*,* European Plaice (*Pleuronectes platessa*), European Flounder (*Platichthys flesus*)*,* Common Dab (*Limanda limanda*).

Location

Baltic Sea (marginal sea in the northeast Atlantic Ocean) including Kattegat, Øresund, western Baltic Sea, and Baltic proper (ICES subdivisions 21-29).

Scale of Analysis

**Spatial extent (lon/lat):** 9.420922, 22.51811, 53.91644, 59.23877 (xmin, xmax, ymin, ymax)

**Spatial resolution:**

1. Approximately 2 nautical miles
   1. Delta Longitude: 3’ 20” or 0.05556 degrees.
   2. Delta Latitude: 2’ or 0.03333 degrees.

**Temporal extent:** 2001-2020

**Temporal resolution:** Yearly, biannual

1. Q1 (Feb-Mar), 2001-2020
2. Q4 (Oct-Nov), 2001-2020

**Boundary:** Polygon, ICES subdivisions 21-29

Biodiversity Data

**Observation Type:** Fisheries-independent demersal trawl survey (DATRAS-BITS).

**Response Data Type:** Catch weight (biomass) density (kg km^-2^).

Predictors

**Types:** Geographic, temporal, oceanographic.

Hypothesis

Raw occurrence data are unable to distinguish correlates to a species' realized niche, where a species achieves positive growth rates and contributes more to ecological functioning. To predict quantitative patterns in demersal fish in the Baltic Sea, seasonally-resolved covariates provide the best predictive quality to approximate a species' realized niche.

Model assumptions:

1. No systematic observation bias.
2. Independence of species observations.
3. Availability of all important predictors; key explanatory variables are available and incorporated in the model.
4. Predictors are free of error.
5. Niche stability/constancy, niche conservatism.

Algorithms

**Modelling techniques:** Three versions of Hierarchical Generalized Additive Models (HGAMs) allowing species-level variation to geographic and seasonal abiotic predictors.

**Model complexity:** Our multi-model approach included an incremental gain in model complexity from simpler geographic-only HGAMs (lon, lat, depth, year) to geographic plus abiotic covariates, and finally including seasonal abiotic covariates. Each model used a zero-inflated distribution assumption (Tweedie) and models were compared using AIC values to identify model parsimony ranks.

**Model averaging:** Model evaluation metrics (discrimination, precision, and accuracy; Waldock *et al.* 2022) are based on average achieved scores in k-fold cross validation. Final predictions are visualized as the difference between average species-specific biomass in the last five years (2016-2020) and the first five years (2001-2005) of the BITS survey. During the revision process, we also compared biomass differences between the first (2001–2010) and last (2011–2020) decades alongside a median rate of change per grid cell (kg km^-2^ yr^-1^). All patterns are calculated within a 0.5° gridcell.

Workflow

**Modelling:** Trained HGAMs based on biomass density, analogous to Species Abundance Models (SAMs; e.g, Waldock *et al.* 2022; Chardon *et al.* 2022), for each demersal fish species hierarchically across the Baltic Sea region (Kattegat, Øresund, western Baltic Sea, Baltic proper). Each model contained a spatiotemporal geographic term to limit autocorrelation and included varying degrees of species-level variation through shared or independent smoothing penalties following Pedersen *et al.* (2019). Each model was evaluated across random k-folds (k = 10). Final predictions for mapping incorporated all observations (Step 5, option 1 in Roberts *et al.* 2017), favoring prediction quality over error estimation although we expect reasonable error estimates from cross validation.

Software

R versions 4.2.1 (R Core Team, 2022); packages ‘stats’ (R Core Team, 2022), ‘raster’ (^a^Hijmans *et al*. 2022), ‘terra’ (^b^Hijmans *et al*. 2022), ‘tidyverse’ (Wickham *et al.* 2019), ‘mgcv’ (Wood, 2023), ‘oce’ (Kelley, 2018), ‘sf’ (Pebesma, 2018), ‘gratia’ (Simpson, 2018)

**Code availability:** <https://github.com/LiamMacNeil/Balt_Demersal_SDMs>

**Data availability:**

1. DATRAS-BITS (Baltic International Trawl Survey) exchange data and swept-area assessment (<https://datras.ices.dk/data_products/download/download_data_public.aspx>).
2. Copernicus marine service environmental layers
   1. [Baltic Sea Physics Reanalysis | Copernicus Marine MyOcean Viewer](https://data.marine.copernicus.eu/product/BALTICSEA_MULTIYEAR_PHY_003_011/description).
   2. [Baltic Sea Biogeochemistry Reanalysis | Copernicus Marine MyOcean Viewer](https://data.marine.copernicus.eu/product/BALTICSEA_MULTIYEAR_BGC_003_012/description).

**Data**

Biodiversity data

**Taxon names:** Atlantic Cod (*Gadus morhua*)*,* Common Dab (*Limanda limanda*)*,* European Flounder (*Platichthys flesus*)*,* European Plaice (*Pleuronectes platessa*).

**Taxonomic reference system:** Linnean

**Ecological level:** Species

**Data sources:** Fisheries-independent demersal trawl survey (Baltic International Trawl Survey; BITS), routinely collected with standardized gears during Q1 (Feb-Mar) and Q4 (Oct-Nov) spanning 2001-2020.

S**ampling design:** Coordinated international demersal trawls (BITS) following ICES (2017) using standardized gears (TVL 930 meshes, TVS 520 meshes) at randomly stratified stations based on ICES subdivisions. Sampling design is described in more detail within ICES (2017).

**Sample Size:** A total of 8991 individual trawls are included.

**Regional mask:** All data are clipped to a boundary region of ICES subdivisions 21-29 and covariates further masked by a buffer polygon 0.5° within the radius of trawl observations.

**Scaling:** Trawl observations were spatially thinned to the resolution of environmental predictors (~2 nautical miles) for each species, during each modelling time window, to reduce the spatial autocorrelation from repeated observations.

**Data cleaning/filtering**:

1. Observations from DATRAS-BITS exchange data and swept area assessments were removed that did not report (1) lon/lat, (2) station number, (3) catch weight (CatCatchWgt), (4) species length class (LngtClass), or (5) door-based swept area (SweptAreaDSKM2; Berg *et al.* 2019).
2. Only trawls designated by DATRAS-BITS as “Valid”, “Additional”, or “Calibration” are considered.
3. Trawls durations below 15 minutes and longer than 90 minutes were excluded.
4. Only observations produced from two standard gear types (TVL 930 meshes, TVS 520 meshes) were included.
5. DATRAS-BITS were trimmed to within 2001-2020, inclusively. The year 2001 represents the time of implementation of standard gear types and the year 2020 represents the final year available for swept area assessments (Berg *et al.* 2019).

| **Background data**: Zeros were imputed for all trawls where our focal species were not recorded, totalling ~22% in frequency.  **Errors and biases:**   1. Coastal regions (e.g., <20 m depth) are poorly covered by the DATRAS-BITS survey, thus we expect poorer prediction accuracy in these areas, otherwise major basins throughout the Baltic Sea (western Baltic Sea, Arkona Basin, eastern Bornholm Basin, and eastern Gotland Basin) are richly covered. 2. Misidentification is expected to be low, given all four species are common focal taxa for demersal surveys. Flounder is a possible exception, because a new Baltic species was only recently demarcated (Momigliano *et al.* 2018), which potentially affected taxonomic identification afterwards in the most northern extent of our study area (Gulf of Riga, and northern Baltic proper) where this species occurs. 3. Data gaps (missing and outliers) for calculating swept area (km^2^; estimate of effort) do exist and are caused predominantly by the lack of gear geometry sensors during deployment (Berg *et al.* 2019). The richest data availability, supporting the most reliable swept area estimates, are provided by Germany, Denmark, and Sweden covering ICES subdivisions 22-26 (western Baltic Sea, Øresund, Arkona Basin, eastern Bornholm Basin). Errors outside these regions (ICES subdivisions 27-29; Baltic proper) are possibly affected by unreliable estimates (AS7).         Data partitioning  **Training data:** Randomized 10-fold cross validation where k-1 folds are iteratively used for model training.    **Validation/Test data:** Performance is assessed for each model on withheld k-fold.    Predictor variables  **Predictor variables:**   1. Physics Reanalysis    1. Sea water potential temperature at sea floor (bottomT).    2. Sea water salinity at sea floor (Sb).      1. Biogeochemistry Reanalysis    1. Mole concentration of dissolved molecular oxygen in sea water at sea floor (O_2_b).     **Data sources:**  Copernicus marine service oceanographic layers   - 1. [Baltic Sea Physics Reanalysis \| Copernicus Marine MyOcean Viewer](https://data.marine.copernicus.eu/product/BALTICSEA_MULTIYEAR_PHY_003_011/description).   2. [Baltic Sea Biogeochemistry Reanalysis \| Copernicus Marine MyOcean Viewer](https://data.marine.copernicus.eu/product/BALTICSEA_MULTIYEAR_BGC_003_012/description).     **Spatial extent (lon/lat):** 9.420922, 22.51811, 53.91644, 59.23877 (xmin, xmax, ymin, ymax)    **Spatial resolution:**   1. Approximately 2 nautical miles    1. Delta Longitude: 3’ 20” or 0.05556 degrees    2. Delta Latitude: 2’ or 0.03333 degrees   **Temporal extent:** 2001-2020  **Temporal resolution:** Yearly, biannual   1. Q1 (Feb-Mar), 2001-2020 2. Q4 (Oct-Dec), 2001-2020   **Coordinate reference system:** longlat; EPSG:4326    **Data processing:** We extracted monthly values for every oceanographic variable and created weighted averages (weighted by observation frequency) for each quarter of each year.    **Errors and biases:** No outstanding errors or biases are expected from data processing.       1. For each reanalysis file (numerical model), a quality information document indicates the following patterns in environmental layers:    1. Bottom oxygen displayed relatively high root-mean-square deviation from observed values in the southern Baltic proper.     **Dimension reduction:** NA  **Model**    Variable pre-selection  **Variable pre-selection:** We focused on bottom-associated variables to pre-select variables which shape environments most often occupied by these demersal fish. Further, we pre-selected core variables (temperature, salinity, oxygen) which have been previously described and attributed to impact Baltic demersal niches (e.g., Rau *et al.* 2012; Smoliński and Radtke, 2017; Orio *et al.* 2019; Brander, 2022).    Multicollinearity  **Multicollinearity:** We assessed variable concurvity– a generalization of collinearity– for each model and found no variable exceeded 0.8. No absolute threshold exists to exclude variables based on concurvity, but GAMs are strongly robust to biases due to concurvity (Wood, 2008).    Model settings  **GAM**: All HGAMs were built in the *bam* function in *mgcv*; all versions shared a Tweedie distribution assumption with a log-link function, plus discretized covariates and fast restricted maximum likelihood (fREML) for efficient computation. Parsimonious model configurations were determined by null space penalization. Variable smoothers were constructed for geographic and abiotic covariates using tensor smooth products for interactions (lon x lat x year; depth x year) species-level variation was incorporated using species-specific smoothing penalties (by = species) in HGAM-II for depth and abiotic covariates. For HGAM version-III (seasonal abiotic covariates), species-level variation was permitted to vary independently across seasons using factor smooths (bs =”fs”). Random intercepts were also included across model versions without factor smooths (HGAM-I, HGAM-II) through random effect smoothers (bs = “re”). For model versions-II (geography + abiotic covariates) and -III (geography + seasonal abiotic covariates), we penalized the square second derivative (*m* = 2) of group-level smoothers to limit excessive curvature (Pedersen *et al.* 2019). To address temporal autocorrelation in model residuals, we added an AR1 autoregressive process based on the observed lag using the *rho* parameter in the *bam* function.  Model estimates  **Coefficients:** Coefficient estimates for GAMs    **Parameter uncertainty:** Coefficient variation within cross-validation.    **Variable importance**: NA    Model selection  **GAM:** Automatic regularization with ‘null space penalization’ (<https://www.rdocumentation.org/packages/mgcv/versions/1.8-42/topics/step.gam>), which penalizes each smoothing term and effectively removes the predictor from model predictions if the smoothing term tends toward zero.    **Model ensembles:** NA    Analysis and Correction of non-independence  **Spatial autocorrelation:** A spatiotemporal model term, varying independently by species, was included in each model to incorporate spatiotemporal dependencies in the data.    **Nested data:** All trawl data were thinned to covariate grid level. Hierarchical groupings including species- and seasonal-level (quarter) variation.    **Assessment**    Performance statistics  Metrics have been chosen from review in Waldock *et al.* (2022)    **Performance on training data:** Pearson’s correlation coefficient (Discrimination), Mean Absolute Error (MAE; Accuracy), dispersion (σ_est_ / σ_obs_)^^[[1]](#footnote-0)^^.    **Performance on validation data:** Pearson’s correlation coefficient (Discrimination), Mean Absolute Error (MAE; Accuracy), dispersion (σ_est_ / σ_obs_).    **Performance on test data:** Pearson’s correlation coefficient (Discrimination), Mean Absolute Error (MAE; Accuracy), dispersion (σ_est_ / σ_obs_).    Plausibility check  **Response shapes:** Partial response curves (Simpson, 2018).    **Prediction**    Prediction output  **Prediction unit:** Species-level catch weight (biomass) density (kg km^-2^).    **Post-processing:** No post-prediction calibration was applied (*sensu* Dormann, 2020) as we do not use conventional classification-based species distribution models with a probabilistic interpretation.  Uncertainty quantification  **Algorithmic uncertainty:** Random cross-validation (k = 10) within training-test set splits where model performance metrics are the averages (± 95 and 5% quantiles). |
| --- |

**References**

Berg, C. W., Brun, M., Börjesson, P., Chaves, C., Degel, H., Lynam, C. P., Martinez, I., Schuchert, P., Soni, V., Velaso, F., Villamor, A., & Wieland, K. (2019). Workshop on Methods to develop a swept-area based effort index (WKSABI). International Council for the Exploration of the Sea (ICES). ICES Scientific Report Vol. 1 No. 3 <https://doi.org/10.17895/ices.pub.4902>.

Brander, K. (2022). Support for the hypothesis that growth of eastern Baltic cod is affected by mild hypoxia. A comment on Svedäng et al. (2022). *ICES Journal of Marine Science*, *79*(7), 2155–2156.<https://doi.org/10.1093/icesjms/fsac070>

Chardon, N. I., Nabe-Nielsen, J., Assmann, J. J., Dyrholm Jacobsen, I. B., Guéguen, M., Normand, S., & Wipf, S. (2022). High resolution species distribution and abundance models cannot predict separate shrub datasets in adjacent Arctic fjords. *Diversity and Distributions*, *28*(5), 956–975.<https://doi.org/10.1111/ddi.13498>.

Dormann, C. F. (2020). Calibration of probability predictions from machine-learning and statistical models. *Global Ecology and Biogeography*, *29*(4), 760–765.<https://doi.org/10.1111/geb.13070>

^a^Hijmans, R.J. (2022). Geographic Data Analysis and Modeling. R package version v. 3.6.14.

^b^Hijmans, R. J., Bivand, R., Forner, K., Ooms, J., & Pebesma, E. (2022). R package version v. 1.7.3. <https://rspatial.org/terra/>

ICES (2017). SISP 7 BITS Manual for the Baltic International Trawls Surveys.<https://doi.org/10.17895/ICES.PUB.2883>

Kelley, D. *“The Oce Package” In Oceanographic Analysis with R*. R package version v. 1.7.10, 91–101 (Springer, 2018).

Momigliano, P., Denys, G. P. J., Jokinen, H., & Merilä, J. (2018). *Platichthys solemdali* sp. nov. (Actinopterygii, Pleuronectiformes): A New Flounder Species From the Baltic Sea. *Frontiers in Marine Science*, *5*, 225.<https://doi.org/10.3389/fmars.2018.00225>

Orio, A., Bergström, U., Florin, A., Lehmann, A., Šics, I., & Casini, M. (2019). Spatial contraction of demersal fish populations in a large marine ecosystem. *Journal of Biogeography*, *46*(3), 633–645.<https://doi.org/10.1111/jbi.13510>

Pebesma, E. (2018). Simple Features for R: Standardized Support for Spatial Vector Data, *The R Journal*, 10(*1*) 439-446. R package version v. 1.0.9.

Pedersen, E. J., Miller, D. L., Simpson, G. L., & Ross, N. (2019). Hierarchical generalized additive models in ecology: An introduction with mgcv. *PeerJ*, *7*, e6876.<https://doi.org/10.7717/peerj.6876>

Rau, A., Lewin, W.-C., Zettler, M. L., Gogina, M., & von Dorrien, C. (2019). Abiotic and biotic drivers of flatfish abundance within distinct demersal fish assemblages in a brackish ecosystem (western Baltic Sea). *Estuarine, Coastal and Shelf Science*, *220*, 38–47.<https://doi.org/10.1016/j.ecss.2019.02.035>

R Core Team (2022). R: A language and environment for statistical computing. R Foundation for Statistical Computing, Vienna, Austria. URL [https://www.R-project.org/](https://www.r-project.org/).

Roberts, D. R., Bahn, V., Ciuti, S., Boyce, M. S., Elith, J., Guillera-Arroita, G., Hauenstein, S., Lahoz-Monfort, J. J., Schröder, B., Thuiller, W., Warton, D. I., Wintle, B. A., Hartig, F., & Dormann, C. F. (2017). Cross-validation strategies for data with temporal, spatial, hierarchical, or phylogenetic structure. *Ecography*, *40*(8), 913–929. <https://doi.org/10.1111/ecog.02881>

Simpson, G.L. (2018). R Package: gratia. Ggplot-based graphics and other useful functions for GAMs fitted using Mgcv, 0.1-0 (Ggplot-based graphics and utility functions for working with GAMs fitted using the mgcv package).

Smoliński, S., & Radtke, K. (2017). Spatial prediction of demersal fish diversity in the Baltic Sea: Comparison of machine learning and regression-based techniques. *ICES Journal of Marine Science*, *74*(1), 102–111.<https://doi.org/10.1093/icesjms/fsw136>

Waldock, C., Stuart‐Smith, R. D., Albouy, C., Cheung, W. W. L., Edgar, G. J., Mouillot, D., Tjiputra, J., & Pellissier, L. (2022). A quantitative review of abundance‐based species distribution models. *Ecography*, *2022*(1), ecog.05694.<https://doi.org/10.1111/ecog.05694>.

Wickham *et al.*, (2019). Welcome to the Tidyverse. Journal of Open Source Software, 4(43), R package version v. 1.3.2 (2022), 1686, <https://doi.org/10.21105/joss.01686>.

Wood, S. N. (2008). Fast Stable Direct Fitting and Smoothness Selection for Generalized Additive Models. *Journal of the Royal Statistical Society Series B: Statistical Methodology*, *70*(3), 495–518.<https://doi.org/10.1111/j.1467-9868.2007.00646.x>

Wood, S. (2023). Package ‘mgcv’. R package version 1.8.41.

Zurell, D., Franklin, J., König, C., Bouchet, P. J., Dormann, C. F., Elith, J., Fandos, G., Feng, X., Guillera‐Arroita, G., Guisan, A., Lahoz‐Monfort, J. J., Leitão, P. J., Park, D. S., Peterson, A. T., Rapacciuolo, G., Schmatz, D. R., Schröder, B., Serra‐Diaz, J. M., Thuiller, W., … Merow, C. (2020). A standard protocol for reporting species distribution models. *Ecography*, *43*(9), 1261–1277.<https://doi.org/10.1111/ecog.04960>.

1. Variance of estimated (σ_est_) and observed (σ_obs_) biomass density [↑](#footnote-ref-0)
